# Honokiol Acts as a Potent Anti-Fibrotic Agent in the Liver through Inhibition of TGF-β1/SMAD Signaling and Autophagy in Hepatic Stellate Cells

**DOI:** 10.3390/ijms222413354

**Published:** 2021-12-12

**Authors:** Seita Kataoka, Atsushi Umemura, Keiichiro Okuda, Hiroyoshi Taketani, Yuya Seko, Taichiro Nishikawa, Kanji Yamaguchi, Michihisa Moriguchi, Yoshihiro Kanbara, Jack L. Arbiser, Toshihide Shima, Takeshi Okanoue, Yoshito Itoh

**Affiliations:** 1 Molecular Gastroenterology and Hepatology, Graduate School of Medical Science, Kyoto Prefectural University of Medicine, 465 Kajii-cho, Kamigyo-ku, Kyoto 602-8566, Japan; s1120@koto.kpu-m.ac.jp (S.K.); k-okuda@koto.kpu-m.ac.jp (K.O.); take1012@koto.kpu-m.ac.jp (H.T.); yuyaseko@koto.kpu-m.ac.jp (Y.S.); taichi@koto.kpu-m.ac.jp (T.N.); ykanji@koto.kpu-m.ac.jp (K.Y.); mmori@koto.kpu-m.ac.jp (M.M.); yitoh@koto.kpu-m.ac.jp (Y.I.); 2 Department of Gastroenterology and Hepatology, Saiseikai Suita Hospital, Suita 564-0013, Japan; kanbara1949d@suita.saiseikai.or.jp (Y.K.); shima0301d@suita.saiseikai.or.jp (T.S.); okanoue@suita.saiseikai.or.jp (T.O.); 3 Department of Dermatology, Emory University School of Medicine, Atlanta, GA 30322, USA; jarbise@emory.edu; 4 Veterans Affairs Medical Center, Decatur, GA 30322, USA

**Keywords:** hepatic fibrosis, stellate cell, honokiol, autophagy

## Abstract

Chronic liver injury may result in hepatic fibrosis, which can progress to cirrhosis and eventually liver failure. There are no drugs that are specifically approved for treating hepatic fibrosis. The natural product honokiol (HNK), a bioactive compound extracted from *Magnolia grandiflora*, represents a potential tool in the management of hepatic fibrosis. Though HNK has been reported to exhibit suppressive effects in a rat fibrosis model, the mechanisms accounting for this suppression remain unclear. In the present study, the anti-fibrotic effects of HNK on the liver were evaluated in vivo and in vitro. In vivo studies utilized a murine liver fibrosis model, in which fibrosis is induced by treatment with carbon tetrachloride (CCl_4_). For in vitro studies, LX-2 human hepatic stellate cells (HSCs) were treated with HNK, and expression of markers of fibrosis, cell viability, the transforming growth factor-β (TGF-β1)/SMAD signaling pathway, and autophagy were analyzed. HNK was well tolerated and significantly attenuated CCl_4_-induced liver fibrosis in vivo. Moreover, HNK decreased HSC activation and collagen expression by downregulating the TGF-β1/SMAD signaling pathway and autophagy. These results suggest that HNK is a new potential candidate for the treatment of hepatic fibrosis through suppressing both TGF-β1/SMAD signaling and autophagy in HSCs.

## 1. Introduction

Chronic liver disease is a major global health issue, causing approximately two million deaths per year worldwide [1,2]. Hepatic fibrogenesis is frequently a critical aspect of chronic liver disease, and it results from long-term, repeated liver injury. These liver injuries occur in a variety of ways, such as chronic alcoholic use, viral infection, metabolic disorders, and autoimmune diseases. When liver injury continues for decades, fibrosis may progress into liver cirrhosis, and eventually serious complications may develop, including liver failure or hepatocellular carcinoma, ultimately leading to death.

Although many advances have been made in understanding the pathogenesis of hepatic fibrosis, there are no specific Food and Drug Administration (FDA)-approved anti-fibrotic drugs and scarcely any effective treatments. The major problem encountered in the treatment of liver fibrosis is the long time scale of progression of hepatic fibrosis and cirrhosis. The chronic mechanisms lead to modified liver vascularization, extracellular matrix composition, and drug metabolism. Current therapies are not sufficient to solve these problems [3], and there remains an urgent need for new effective and safe therapies.

To date, the most effective approach to prevent and treat liver fibrosis is to eradicate hepatitis viruses or to remove or ameliorate other causative agents, such as abstinence from alcohol in the case of alcoholic liver diseases. However, such treatments remain unrealistic for patients with liver fibrosis due to other causes, such as genetics, autoimmune liver diseases, or non-alcoholic steatohepatitis (NASH).

As activated hepatic stellate cells (HSCs) play a central role in hepatic fibrogenesis and the progression of fibrosis [4], they represent a potential general therapeutic target. In normal liver tissues, HSCs are quiescent; however, in injured liver tissues, quiescent HSCs transdifferentiate into activated HSCs, which exhibit a myofibroblast-like phenotype and generate cytokines and growth factors, such as transforming growth factor-β1 (TGF-β1) [5]. Sustained HSC activation and proliferation lead to excessive accumulation of extracellular matrix components. Therefore, inhibition of the accumulation of activated HSCs by modulating either their activation or proliferation is a potential therapeutic strategy for the resolution and the treatment of liver fibrosis. While the precise mechanisms by which HSC activation is involved in the progression of chronic liver injury to cirrhosis have not been fully elucidated, it is likely that increased autophagic flux plays a key role [6,7,8], so that partial inhibition of HSC activation by autophagy inhibitors unveils a potential new therapeutic strategy for liver fibrosis.

As almost all cirrhotic livers arise from chronic and common liver diseases, including NASH, the effective treatment and prevention of hepatic fibrosis should rely on the application of safe and low-cost drugs, including naturally occurring compounds. In this study, then, we evaluated the anti-fibrotic activity of honokiol (HNK), a natural product, both in vivo in a mouse model of fibrosis and in vitro in HSCs, and we investigated the underlying mechanism of this activity. HNK (3,5-di-(2-propenyl)-1,1-biphenyl-2,2-diol), is a low molecular weight biphenolic compound isolated from *Magnolia grandiflora* [9,10], and it exhibits a variety of pharmacological effects, including antioxidant, anti-inflammatory, and anti-tumor effects [11]. HNK has also been reported to have anti-fibrotic properties [12,13,14], but its effects on the progression of fibrosis in mouse models or the status of autophagy in HSCs have not been thoroughly examined. Here, we demonstrate that HNK exerts anti-fibrotic properties in a carbon tetrachloride (CCl_4_)-induced mouse fibrosis model and that it reduces HSC activity by inhibiting both the TGF-β1/SMAD signaling pathway and autophagy.

## 2. Results

### 2.1. HNK Treatment Attenuates Fibrosis Development in Mice with CCl_4_-Induced Liver Injury

To treat or prevent fibrosis progression in a chronically injured liver, non-toxic naturally occurring compounds or herbal drugs are likely to be the most suitable for long-term use. To that end, we focused on HNK, a bioactive compound extracted from the tree *Magnolia grandiflora* that has been widely used as a constituent of herbal anti-tumor drugs [11,15]. It has also been reported that HNK exhibits suppressive effects in a rat liver fibrosis model [12,13]; however, the mechanisms of action remain unclear, and its effect on the mouse liver has not yet been explored.

**Figure 1:**
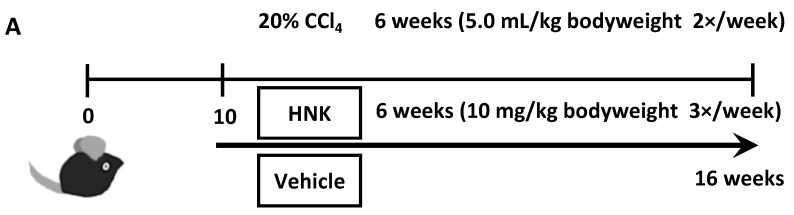

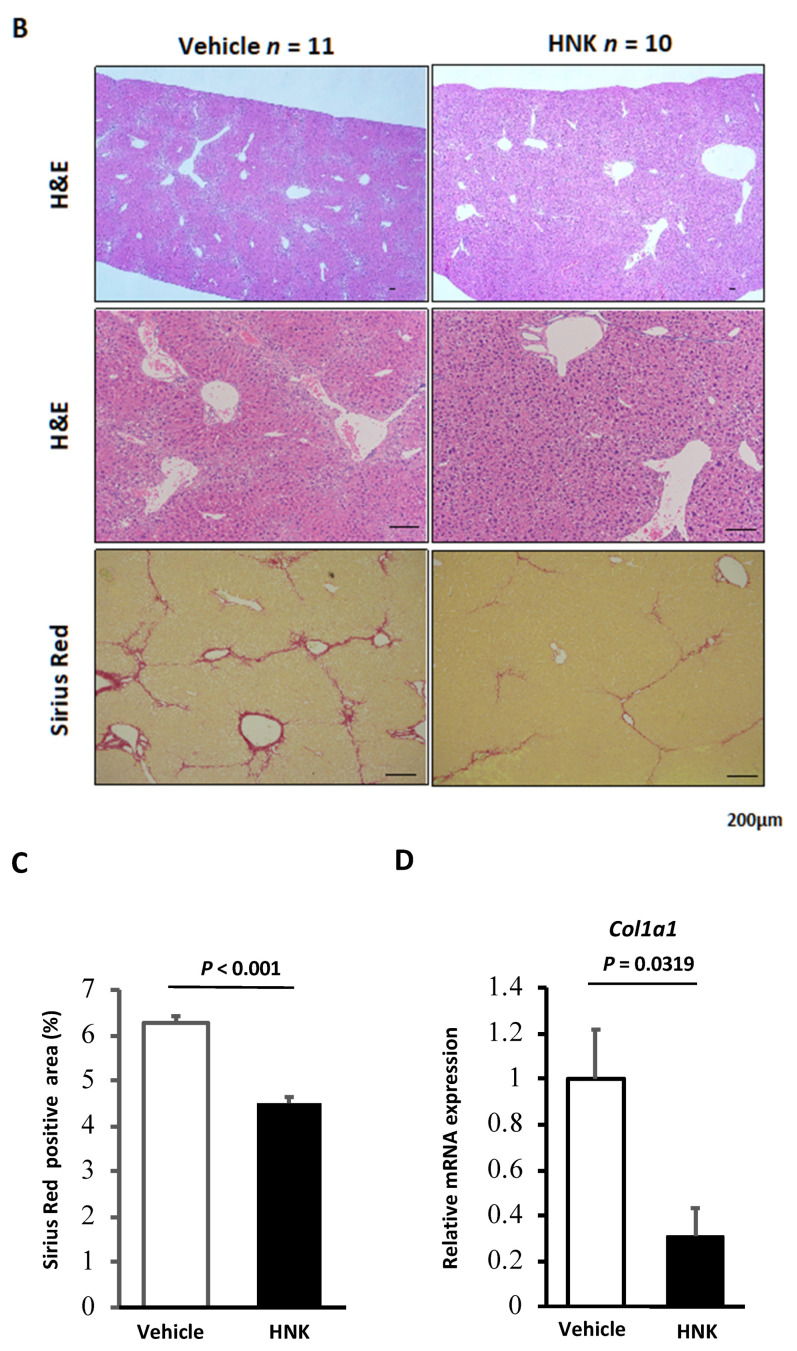
Honokiol (HNK) treatment attenuates fibrosis development in mice with CCl_4_-induced liver injury. WT mice were injected with 20% CCl_4_ at 5.0 mL/kg body weight twice a week for 6 weeks (from 10 to 16 weeks of age). HNK was dosed at 10 mg/kg body weight or vehicle control administered three times per week on a day without CCl_4_ injection for the same six weeks. Liver injury and fibrosis were assessed by H&E staining, Sirius-Red staining, and mRNA expression levels. (**A**) Schematic of CCl_4_-induced mouse liver injury model. (**B**) Representative microphotographs of liver sections stained with HE and Sirius Red. The bottom row of HE stained images are enlarged images of the same specimens as the top row, respectively. Scale bars represent 200 μm. (**C**) Sirius Red positive areas (%) quantified by BZ-X800 Analyzer. (**D**) Relative mRNA expression levels of *Col1a1* in mouse liver. *Gapdh* was used as an internal control for real-time quantitative PCR. All graphs represent the mean ± SD.

In the present study, we examined the ability of HNK to inhibit fibrosis progression in the livers of mice treated with CCl_4_ from 10 to 16 weeks of age to explore its anti-fibrotic effects. The CCl_4_-induced liver fibrosis model has been widely used in liver fibrosis and cirrhosis research because of its high reproducibility and similarity to human liver fibrosis and cirrhosis [16]. In previous reports, administration of HNK to mice for the treatment of cancer was conducted at doses ranging from 0.5 to 150 mg/kg [11]. In this study, we adopted a relatively low concentration, 10 mg/kg, to reduce toxicity, since HNK is intended to be used as a preventive and ameliorative agent for fibrosis, not as an anticancer agent. When we administered HNK or vehicle via intraperitoneal (IP) injection into mice simultaneously being treated with CCl_4_ (Figure 1A), mice in both groups developed typical fibrosis at 16 weeks of age as expected (Figure 1B). Notably, HNK treatment significantly reduced fibrosis with collagen fiber deposition as demonstrated by HE and Sirius-Red staining and by analysis of the expression of the profibrogenic gene collagen type I alpha-1 chain (*Col1a1)* in the liver (Figure 1C,D). These results suggest that HNK exerts anti-fibrotic effects in the mouse model and attenuates fibrosis development.

Neither HNK nor vehicle induced obvious liver injury as detected by HE staining in mice that received oil without CCl_4_ (HNK with oil group and vehicle with oil group) (Figure S1A). In addition, there were no significant differences in levels of alanine aminotransferase (ALT) between HNK and vehicle treated groups in either the CCl_4_ model or the oil control (Figure S1D). Body weight at the time of analysis was also not significantly different among the four groups (Figure S1E). These results suggest that HNK administration does not cause significant liver injury or apparent side effects. Moreover, HNK reduced the number of F4/80-positive macrophages in the CCl_4_-treated mouse liver (Figure S1G,H). HNK administration may attenuate liver damage through a mechanism that is different from the one by which HNK inhibits the progression of fibrosis.

### 2.2. HNK Treatment Suppresses HSC Activation in the CCl_4_-Induced Fibrosis Mouse Model

**Figure 2:**
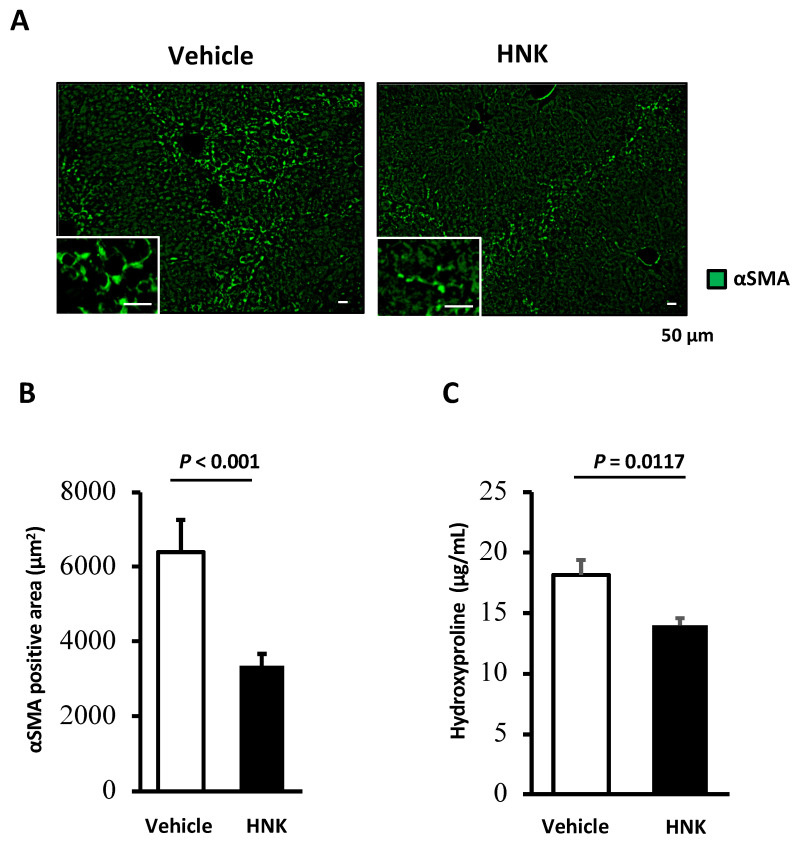
HNK treatment suppresses HSC activation in the livers of mice treated with CCl_4_. Fibrogenesis in mice treated with CCl_4_ with HNK (*n* = 4 per group) were assessed by immunofluorescence (IF) and hydroxyproline assays. (**A**) α-Smooth muscle actin (αSMA) protein (Green). (**B**) αSMA positive area (μm^2^) quantified by BZ-X800 Analyzer. (**C**) The results of the hydroxyproline assay. Scale bars represent 200 μm. All graphs represent the mean ± SD.

Since HSCs are known to play a central role in hepatic fibrosis, we focused on analyzing these cells in fibrotic mouse liver. Immunofluorescent staining of α-smooth muscle actin (αSMA), a representative marker of activated HSCs, was performed. The vehicle treatment group showed abundant αSMA-positive areas; on the other hand, HNK treatment was associated with a significant reduction in αSMA-positive areas (*p* < 0.001) (Figure 2A,B). Analysis of hydroxyproline content also demonstrated that HNK treatment significantly reduced the deposition of collagen fibers in the liver (Figure 2C). These findings suggest that HNK can at least partly attenuate hepatic fibrosis though deactivating HSCs. In addition, HNK treatment reduced SMAD2/3-positive activated HSCs in livers of CCl_4_-treated mice (Figure S1B,C). This latter finding is of particular interest, in that TGF-β1 is considered to be a master profibrogenic cytokine and a promising target in the treatment of fibrosis, and SMAD2/3 are known to act as intracellular mediators in the TGF-β1 signaling pathway [17]. These observations demonstrate that HNK attenuates fibrosis progression by suppressing activated HSCs in this mouse model.

### 2.3. HNK Treatment Suppresses Proliferative and Fibrogenic Activities in Human Activated Hepatic Stellate Cells

From the findings in the mouse model, we concluded that HNK can attenuate hepatic fibrosis by downregulating HSCs. To examine the direct effects and the mechanism underlying the anti-fibrotic properties of HNK on HSCs, we performed an in vitro study by analyzing LX-2, a human activated hepatic stellate cell line. We determined appropriate concentrations of HNK to use by reference to previous reports. For example, HNK has been used at concentrations ranging from 0 to 150 µM in cancer cell lines from various organs [11].

**Figure 3:**
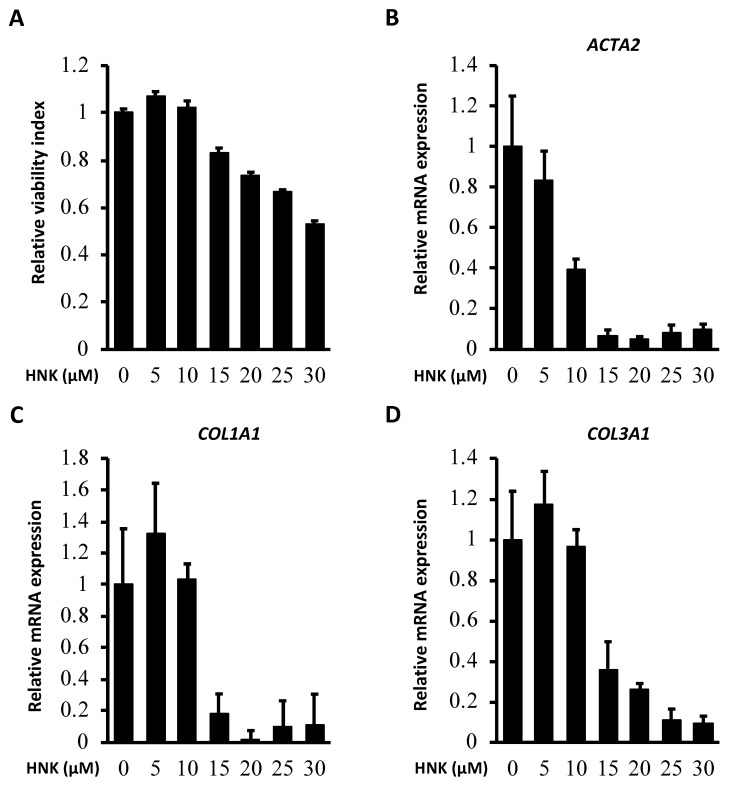
HNK treatment suppresses proliferative and fibrogenic activities in human activated hepatic stellate cells. Effect of HNK on proliferation and fibrogenesis of the HSC line LX-2. (**A**) Cell viability and proliferation of LX-2 cells after a 24 h incubation with the noted concentrations of HNK as determined by the WST8 method. (**B**–**D**) Relative expression of *ACTA2*, *COL1A1,* and *COL3A1* in LX-2 cells 24 h after incubation with the indicated concentrations of HNK. *GAPDH* was used as an internal control for reverse transcription-quantitative PCR. All graphs represent means ± SD.

**Figure 4:**
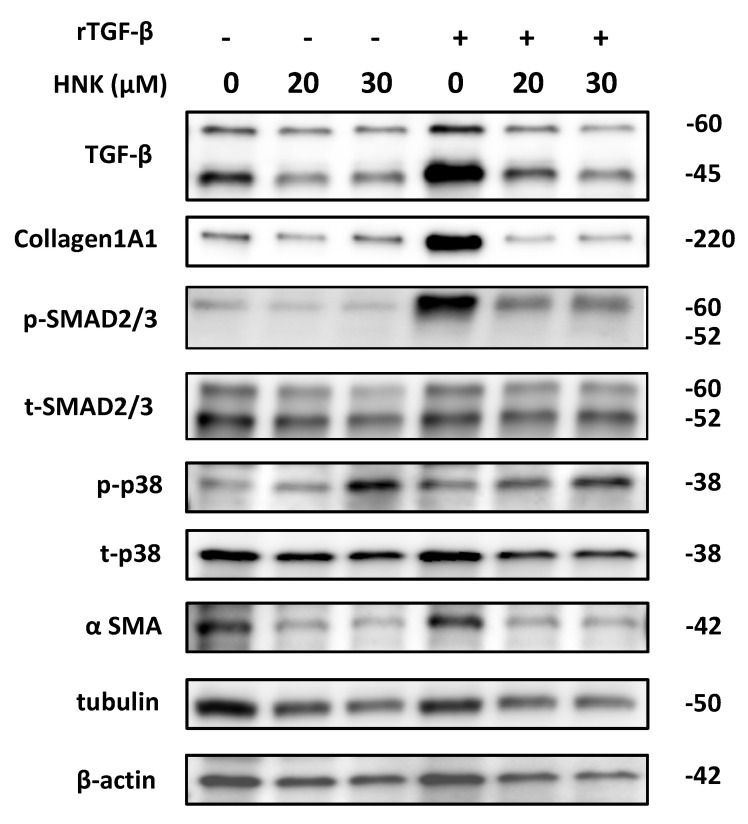
HNK suppresses TGF-β1 signaling pathways in LX-2 cells. Immunoblot analysis of TGF-β1 signaling, MAPK signaling, mTOR signaling, and fibrogenesis pathways in LX-2 cells 24 h after incubation in the presence of HNK at the noted concentrations for 24 h, with or without rTGF-β1 (10 ng/mL) treatment. Tubulin and β-actin were used as loading controls. Numbers on the right indicate the noted molecular weights (kDa). Phosphorylated SMAD2/3 (p-SMAD2/3); total SMAD2/3 (t-SMAD2/3); phosphorylated p38 (p-p38); total p38 (t-p38).

The cell viability/proliferation assay and the TUNEL assay revealed that HNK efficiently suppressed LX-2 proliferation in a dose-dependent manner (Figure 3A) without an increase in the rate of cell death (Figure S2A,B). HNK also reduced the expression of mRNAs for αSMA (*ACTA2*) and collagen components (*COL1A1* and collagen type III alpha-1 chain (*COL3A1*)) (Figure 3B–D). Notably, HNK also suppressed TGF-β1 signaling in LX-2 cells as stimulated by recombinant TGF-β1 (rTGF-β1) (Figure 4 and Figure S4A). Stimulation with rTGF-β1 increased the expression of collagen at the protein level and the phosphorylation of SMAD (Figure 4 and Figure S4A). HNK treatment clearly suppressed collagen expression and the phosphorylation of SMAD, consistent with prevention of HSC activation by attenuation of TGF-β1/SMAD signaling pathways.

### 2.4. HNK Suppresses Autophagy in LX-2 Cells

It is reported that in mice, induction of liver injury with CCl_4_ increased levels of autophagy, and within injured human liver tissue, features of autophagy in activated stellate cells have been observed [7]. In addition, autophagy is known to regulate HSC activation in the progression of liver fibrosis [6].

**Figure 5:**
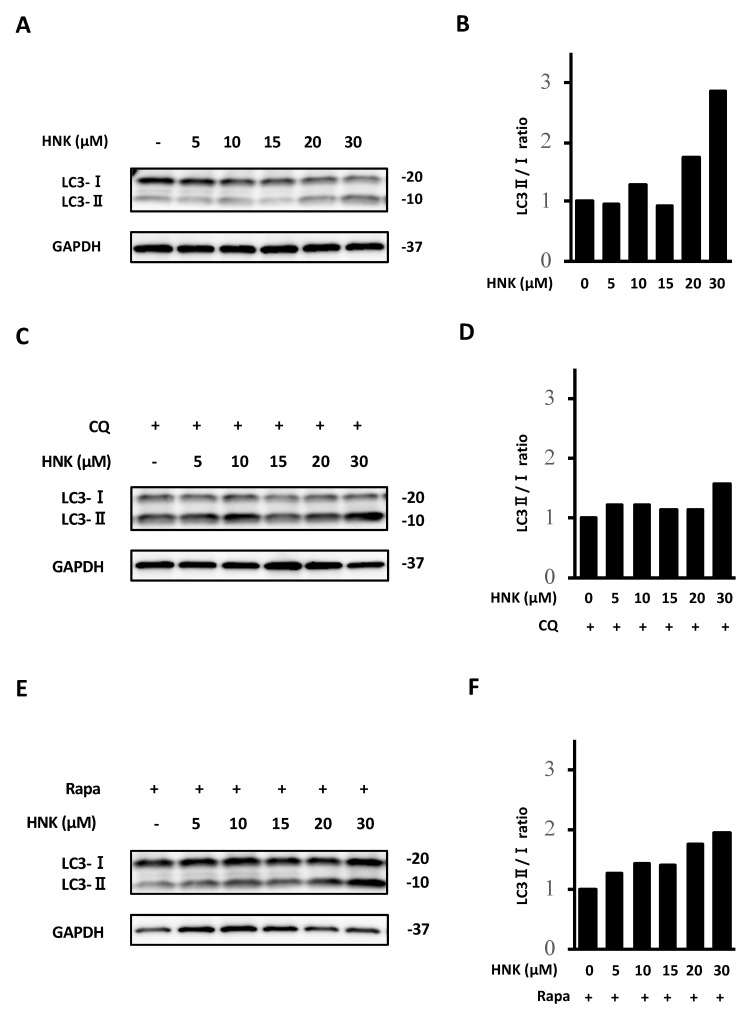

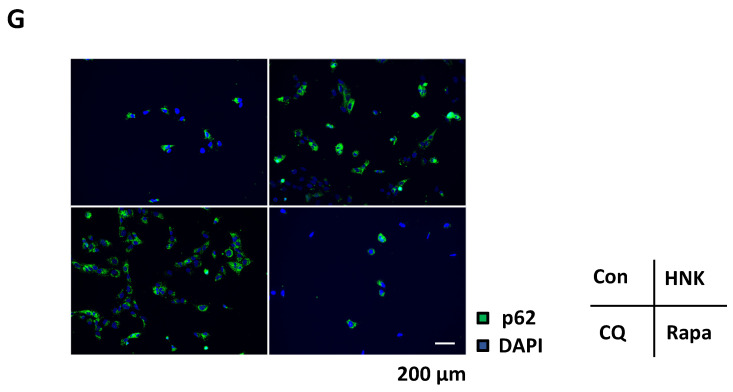
Effects of HNK in suppressing LX-2 activation related to autophagy pathway. (**A**–**F**) Immunoblot of markers related to autophagy (LC3-I, -II) in LX-2 cells incubated with the noted concentrations of HNK with or without 10 μM chloroquine phosphate (CQ) or 2.5 μM rapamycin (Rapa). GAPDH was used as a loading control. Numbers on the right indicate the noted molecular weights (kDa). The ratio of LC3-II to LC3-I (LC3-II/LC3-I) in each group is shown as fold change with reference to the vehicle-treated group. (**G**) Immunofluorescence analysis (IF) of p62 protein (green) in LX-2 cells counterstained with DAPI (blue) after 12 h incubations with DMSO (negative control), 20 μM HNK, 10 μM CQ (without HNK), or 2.5 μM Rapa (without HNK). Scale bars represent 200 μm.

HNK has been reported to affect autophagy in multiple cells and organs [18,19]; however, there is no report regarding this effect in HSCs. Therefore, we evaluated the autophagy flux of LX-2 cells upon HNK treatment. HNK treatment increased the LC3II/I ratio in a dose dependent manner (Figure 5A,B and Figure S4A,B). By contrast, HNK did not affect the LC3II/I ratio when the autophagy inhibitor chloroquine phosphate (CQ) was included in the incubation (Figure 5C,D and Figure S4C). Next, we used rapamycin (Rapa) to enhance autophagy in LX-2 cells and found that HNK increased the LC3II/I ratio in a dose dependent manner (Figure 5E,F and Figure S4D). Furthermore, HNK treatment increased aggregation of p62 as evaluated by immunofluorescence analyses (Figure 5G). These results suggest that HNK suppresses autophagy in LX-2 cells.

The administration of CQ alone inhibited the expression of αSMA in LX2 cells, and the effect of HNK on this LX2 activation seemed to be attenuated when HNK and CQ were co-administered (Figure S3). This suggests that CQ inhibits LX2 activation via inhibition of autophagy, thus abrogating the effect of HNK on the inhibition of activation of LX2. These findings suggest that HNK can suppress autophagy flux in human HSC cells.

### 2.5. HNK Suppresses mTOR Signaling, but Activates p38

**Figure 6:**
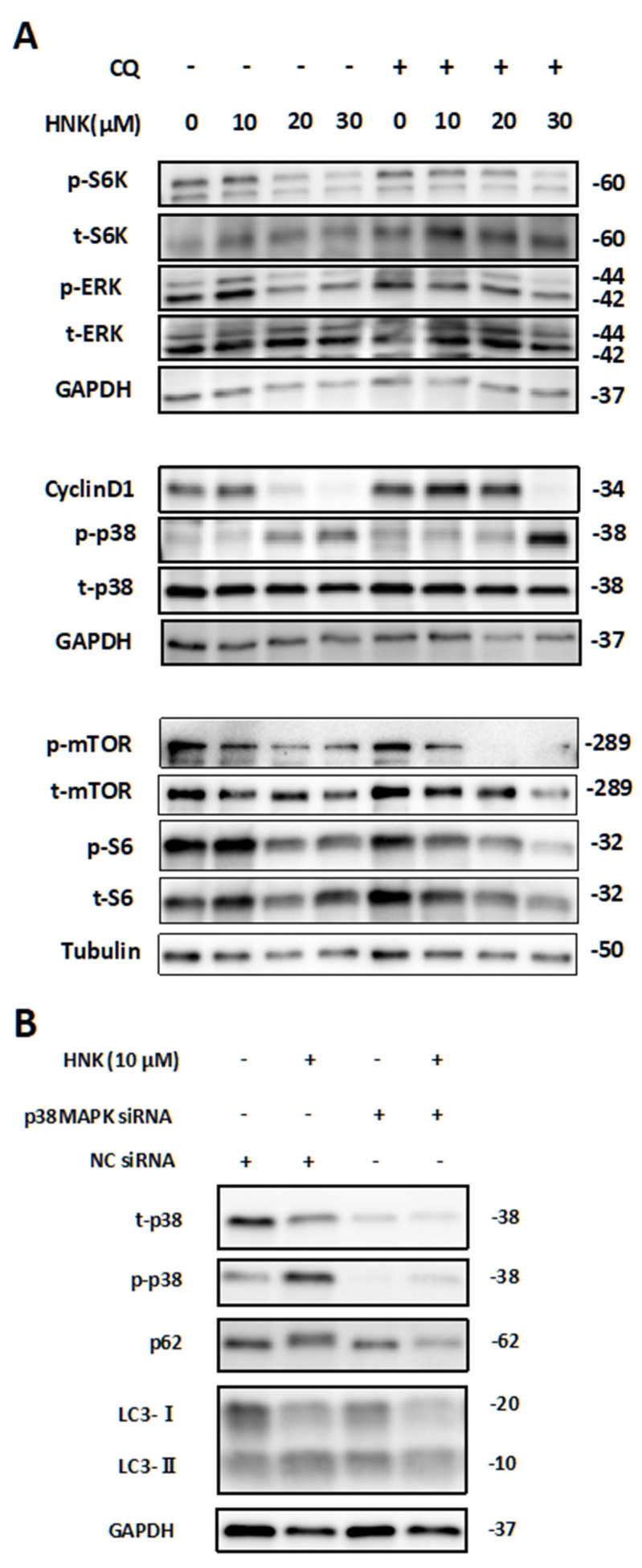

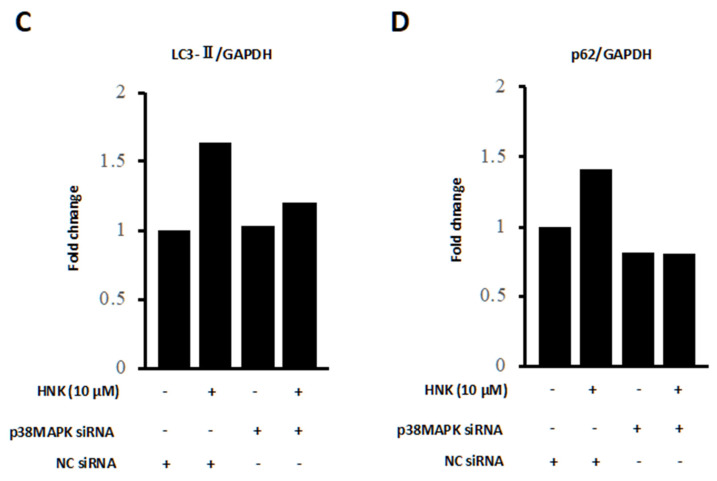
Assessment of the effect of HNK on signaling cascades associated with autophagy inhibition in HSC. (**A**) Immunoblot analysis of mTOR signaling and MAPK signaling in LX-2 cells 24 h after incubation in the presence of HNK at the noted concentrations for 24 h, with or without 10 μM CQ treatment. (**B**) Immunoblot analysis was performed to detect levels of p-p38 MAPK, t-p38 MAPK, LC3, and p62 in LX-2 cells transfected with p38 MAPK or negative control (NC) siRNA and further treated with HNK for 24 h. (**C**,**D**) To compare ratios of LC3-II or p62 to GAPDH across samples, the fold change was calculated by dividing the normalized expression from each lane by the normalized expression of the control sample (HNK 0, with NC siRNA). Numbers on the right indicate the noted molecular weights (kDa). Phosphorylated S6K (p-S6K); total S6K (t-S6K); phosphorylated S6 (p-S6); total S6 (t-S6); phosphorylated ERK (p-ERK); total ERK (t-ERK); phosphorylated p38 (p38); total p38 (t-p38).

Next, we analyzed signaling cascades other than TGF-β1 signaling that are involved in HSC activation. As shown in Figure 4, HNK treatment was found to decrease the basal and TGF-β1-stimulated phosphorylation of mTOR. In addition, HNK treatment suppressed mTORC1 signaling, as indicated by decreased phosphorylation of S6K and S6, ERK phosphorylation, and cyclin D1 levels (Figure 6A). By contrast, we found that HNK treatment resulted in increased activation of p38 MAPK (Figure 6A and Figure S4E), which is known to mediate autophagy inhibition [20,21,22]. This p38 activation may account for one of the mechanisms of autophagy inhibition by HNK as shown in this study. To further investigate whether this mechanism is also observed in LX2 cells, we evaluated the autophagy pathway by administering HNK to LX2 cells transfected with p38 MAPK siRNA. Here, the increases of both LC3-II and p62 caused by HNK administration were attenuated in the p38 MAPK siRNA group (Figure 6B–D and Figure S4F). These results suggest that HNK treatment downregulates HSCs via inhibition of TGF-β1 signaling and autophagy flux via p38 activation.

## 3. Discussion

Molecular mechanisms of liver fibrosis have been extensively investigated in the search for novel anti-fibrotic therapies. Clinical trials testing drugs which potentially improve or prevent fibrosis have been conducted, but no drugs have been approved yet [23]. Activation of HSCs, i.e., the trans-differentiation of quiescent, vitamin-A-storing cells into proliferative, fibrogenic myofibroblasts, is now well established as a central driver of fibrosis in experimental and human liver injury [3]. Notably, autophagy of activated stellate cells is required for hepatic fibrogenesis in mice [6,7]. An increased autophagic flux during HSC activation suggested that autophagy participates in HSC trans-differentiation into a myofibroblastic phenotype [6]. Another report suggested that autophagy provides energy that is necessary to support stellate cell activation through lipid droplet mobilization, liberation of free fatty acids, and mitochondrial β-oxidation [7]. These events allow the activated cell to maintain energy homeostasis in the face of increasing cellular energy demands conferred by fibrogenesis and proliferation.

HNK is an attractive prospect for alleviation of the factors that lead to liver damage. It is a low molecular weight biphenolic natural product that has been shown to have a variety of pharmacological actions, including anti-tumor, anti-inflammatory, anti-oxidant, and anti-angiogenic effects [11,24]. It has been reported that HNK treatment reduces histological liver damage and blunts the elevation in tissue of levels of inflammatory cytokines in rats treated with CCl_4_ and concanavalin A (Con A), respectively [12,13]. In our study, HNK reduced the number of F4/80-positive infiltrating macrophages, indicating that HNK attenuated inflammatory liver damage caused by CCl_4_ (Figure S1G,H). It is possible that factors that are potentially induced by macrophages and that activate HSCs, such as TGFβ, PDGF, TNF, IL1β, MCP1, CCL3, and CCL5, are reduced [3], which may result in the suppression of HSC activation. This would represent one mechanism by which HNK affects the reduction of liver fibrosis.

Furthermore, the inhibition of fibrosis by HNK has been shown to occur through inhibition of the TGF-β1 signaling pathway in lungs and kidneys, resulting in improvement in the fibrosis condition [25,26]. In both CCl_4_ and Con A models in rat, HNK attenuated the induced liver fibrosis via restoring antioxidant defense mechanisms, downregulating inflammatory cascades and inhibiting TGF-β1/SMAD/MAPK signaling pathways [12,13]. Although several reports have demonstrated an effect of HNK on inhibiting liver fibrosis [12,13], the underlying antifibrotic mechanisms, especially associated with hepatic stellate cells, are not fully understood. Interestingly, a fungal toxin, gliotoxin, which induces apoptosis in HSCs, enhances the resolution of fibrosis in livers treated with CCl_4_, suggesting that an inhibition of fibrogenesis may protect against hepatic necrosis in inflammatory liver damage [27]. When we examined the direct effect of HNK on LX-2 HSCs, we found that HNK reduced the activity of these HSCs, even in the absence of immune cells. These results suggest that HNK not only acts on HSCs through immune cells, but also acts directly on HSCs. Thus, we decided to focus on HSCs as a promising potential target of HNK among liver-resident cells, including inflammatory cells.

Lee et al. reported that HNK exerts apoptotic and antifibrotic effects via activation of GSK3β and inhibition of Wnt3a/β-catenin signaling pathway in hepatic stellate cells [14]. A major transforming growth factor, TGF-β1 has long been implicated in liver fibrosis [28]. TGF-β1 is maintained in an inactive state by binding to a latency-associated peptide. When activated, TGF-β1 exerts its biological and pathological activities via the SMAD signaling pathway, which mediates collagen synthesis and plays an important role in the development of liver fibrosis [17,28]. Our study showed that HNK suppressed fibrosis progression by inhibition of the TGF-β1/SMAD signaling pathway in HSC cells. These results suggest that inhibition of TGFβ1/SMAD signaling may be one of the main mechanisms underlying the anti-fibrotic effect of HNK.

We showed that HNK suppressed autophagy in HSC. Selective reduction of autophagic activity in stellate cells can be a potential therapy for patients with fibrotic diseases, including liver fibrosis [6,7,8]. Thoen et al. reported that increased autophagic flux was observed when cultured mouse HSCs were activated, and that the treatment of both mouse and human HSCs with the autophagy inhibitor bafilomycin A1 suppressed the expression of profibrotic markers and cell proliferation in HSCs [6]. Hernandez-Gea et al. also suggested that loss of autophagic function by 3-methyladenine or CQ reduced fibrogenesis and matrix accumulation by inhibiting stellate cell activation through lipid droplet mobilization, liberation of free fatty acids, and mitochondrial β-oxidation in the cultured mouse HSCs and in the mouse liver [7]. It is also reported that HNK induces autophagy in glioma [29], neuroblastoma [30], squamous cell carcinoma [31], and lung cancer cells [32], and this induction leads to tumor suppression mainly due to triggering apoptosis. Apart from cancer treatment, HNK is also known to suppress autophagy and pulmonary arterial hypertension [19]. Our study suggests that HNK administration acts in a direction that inhibits autophagy in HSCs. Inhibition of autophagy in HSCs has been reported to inhibit fibrosis as described above, suggesting that suppression of autophagy is one of the mechanisms by which HSC activation is inhibited by HNK.

We next investigated the factors that inhibit autophagy by HNK in HSC and observed the activation of p38 MAPK. It has been reported that administration of HNK to HepG2, a hepatocellular carcinoma cell line, suppressed proliferation via activation of p38 MAPK, but this report did not investigate the relationship between p38 MAPK and autophagy [33]. It was also suggested that HNK treatment of glioblastoma cell lines induced apoptosis via activation of p38 MAPK, but this study also did not discuss the relationship between p38 MAPK and autophagy [34]. On the other hand, in breast cancer cells, the β-carboline alkaloid flavopereirine blocked autophagy through nonclassical p38 MAPK signaling [20]. Flavopereirine activates p38 MAPK, which in turn suppresses autophagy independent of mTORC1. In research on microglia, activation of p38 MAPK has been also shown to inhibit autophagy by reducing the activity of ULK1 [21]. Furthermore, in senescent human CD8+ T cells, p38 signaling inhibits autophagy in an mTOR-independent manner [22]. As described above, p38 activation suppresses autophagy through a pathway independent of mTORC1. Accordingly, HNK-mediated suppression of mTORC1- and p38 MAPK-independent autophagy was observed in this study. In these experiments, transfection of LX2 with si-p38MAPK attenuated the effect of HNK on autophagy suppression. Thus, p38 MAPK-mediated activation of HSCs by HNK may play a pivotal role in attenuating liver fibrosis via suppression of autophagy. To the best of our knowledge, this is the first study to clarify the precise mechanism of the anti-fibrotic effects of HNK on HSCs, including inhibition of autophagy.

In summary, we demonstrated that the natural product HNK suppresses the progression of fibrosis in a mouse model. Mechanistically, HNK suppressed TGF-β1/SMAD signaling pathway and autophagy in HSCs. Our results also suggest that HNK attenuates HSC activation via autophagy suppression, at least partially. Interestingly, HNK induced p38 activation, which is known to downregulate autophagy, and it may play a pivotal role in attenuation of HSC activation in a TGF-β1/SMAD signaling pathway-independent manner. Notably, no FDA-approved anti-fibrotic drugs are available, and the effective treatment and prevention of hepatic fibrosis should rely on the application of safe and low-cost drugs. HNK is reported to possess anti-tumor properties as well as suppressive effects on fibrosis in the liver. Since HNK has been widely used as a constituent of herbal drugs in clinical practice, it has therapeutic potential for clinical applications for the treatment of hepatic fibrosis and for the prevention of liver cirrhosis and liver cancer. Thus, further studies in this regard are expected to validate its disease prevention and therapeutic mechanisms in humans.

## 4. Materials and Methods

### 4.1. Animals

All animal studies were performed in accordance with the guidelines for the care and use of live animals of the Kyoto Prefectural University of Medicine (KPUM; Kyoto, Japan) and were approved by the KPUM Institutional Animal Care and Use Committee (#M2020-109, -146, -149, and -510). C57BL/6J mice were purchased from SHIMIZU laboratory Supplies Co., Ltd. All mice used in this study were male and were maintained in filter-topped cages under a 12 h/12 h dark/light cycle at KPUM. Mice were fed a diet of food and water that were sterilized by autoclaving.

To generate mice with CCl_4_-induced chronic liver inflammation, 20% CCl_4_ (Sigma-Aldrich, St. Louis, MO, USA) was prepared by dissolving 2 mL of CCl_4_ in 8 mL olive oil (Wako, Osaka, Japan). This solution was injected IP at a dose of 5.0 mL/kg bodyweight into 10-week-old male mice, twice per week for six weeks. To investigate the effects of HNK with non-CCl_4_-treated mice, a group of mice treated with olive oil not containing CCl_4_ was also generated at the same time as the CCl_4_-treated group.

The mice were divided randomly into four groups to be treated with honokiol or vehicle. HNK was dissolved in PBS with 20% Intralipid^®^ (Sigma-Aldrich, St. Louis, MO, USA) and prepared at a concentration of 10 mg/kg bodyweight immediately before injection into mice. The vehicle control consisted of PBS with 20% Intralipid^®^. Treatment with HNK or vehicle began simultaneously with CCl_4_ or control oil treatment. HNK or vehicle was delivered IP three times per week for six weeks (HNK and CCl_4_ group: n = 10; vehicle and CCl_4_ group: n = 11; HNK and oil group: n = 5; vehicle and oil group: n = 5). The 16-week-old mice were sacrificed after the treatment was complete.

Liver tissues were fixed in 10% formalin, embedded in paraffin, sectioned, and either stained with hematoxylin and eosin (HE) or processed for Sirius-Red staining and immunofluorescence analysis as previously described [35]. Details are provided in Supplementary Materials and Methods. The mouse liver specimens were stained with HE and Sirius-Red, then evaluated independently by two well-trained hepatologists at KPUM who were blinded to other analyses. Histological analyses, including investigations of inflammation, necrosis or cellular injury, fibrosis, steatosis, cholestasis, vascular injury, and other characteristics, were performed with reference to the literature [36]. A BZ-X800 microscope and its analyzer (Keyence Corporation, Osaka, Japan) were used to assess HE and Sirius Red staining and areas of fluorescence. The Sirius Red-positive areas (expressed as percentage) in all pathological sections were calculated. For immunofluorescence staining, 10 randomly selected fields of view at 200-fold magnification were used, and the total absolute value of the colored area (μm^2^) was averaged.

### 4.2. Human HSC Cells

Cells of the immortalized HSC cell line LX-2 were purchased from Merck Millipore (SCC064; Burlington, MA, USA). LX-2 cells were cultured in high glucose Dulbecco’s modified Eagle’s medium (Life Technologies, Carlsbad, CA, USA) supplemented with 1% L-glutamine (Life Technologies, Carlsbad, CA, USA), 1% penicillin-streptomycin (Dutscher, Bruxelles, Belgium), and 10% fetal bovine serum (HyClone, Logan, UT, USA). Cells were maintained at 37 °C in the presence of 5% CO_2_ in a humidified incubator. Treatments were performed with the indicated concentrations of HNK for the specified time periods. Relative cell viability and proliferation was determined via the WST-8 colorimetric method using Cell Count Reagent SF (Nacalai Tesque, Kyoto, Japan).

### 4.3. Immunofluorescence Analysis

Immunofluorescence analyses were performed on liver tissues and LX-2 cells treated with HNK or vehicle control essentially as described in Supplementary Materials and Methods. Briefly, cells were incubated on a glass chamber slide with the indicated drug, covered, and incubated with ice-cold 100% methanol for 10 min at −20 °C. After PBS washes, cells were blocked with diluted donkey serum for 30 min at room temperature. Rabbit anti-αSMA (DAKO Agilent Technologies, Santa Clara, CA, USA), rabbit anti-p62 (Progen, Heidelberg, Germany), rabbit anti-SMAD2/3 (Cell Signaling Technology, Danvers, MA, USA), and Alexa Fluor 488-conjugated donkey anti-rabbit IgG (Jackson ImmunoResearch Inc., West Grove, PA, USA) antibodies were used as the primary and secondary antibodies, respectively. Detailed information about antibodies used is available in the Supplementary Materials and Methods section. After washing with PBS, slides were mounted with medium containing DAPI (Vector Laboratories, Burlingame, CA, USA). A BZ-X800 microscope was used for immunofluorescence analyses.

### 4.4. RNA Isolation and Quantitative RT-PCR

LX-2 cells were incubated for 24 h with the indicated concentrations of HNK. We extracted and purified RNA using TRIzol (Thermo Fisher Scientific, Pittsburgh, PA, USA) and chloroform and isopropanol. RNA (1 μg) was reverse-transcribed to generate cDNA using a PrimerScript RT cDNA Synthesis Kit (Takara Bio, Shiga, Japan). Individual gene expression was quantified by real-time qPCR using SYBR FAST qPCR Master Mix (Kapa Biosystems, Wilmington, MA, USA) and a LightCycler 96 Real-Time PCR system (Roche Diagnostics, Mannheim, Germany). Gene expression was normalized to the expression of a housekeeping control gene (*GAPDH* or *GUSB*). The primers used for real-time qPCR analyses are listed in the Supplementary Materials and Methods section (Table S1).

### 4.5. Immunoblot Analysis

Human Recombinant TGF-β1 (PeproTech Inc., Cranbury, NJ, USA) was used to activate HSC cells. CQ (Sigma-Aldrich, St. Louis, MO, USA) and Rapa (Thermo Fisher Scientific, Pittsburgh, PA, USA) were used to evaluate autophagy flux. Following treatment, liver samples and LX-2 cells were homogenized in RIPA buffer, and then equal amounts of liver homogenates were fractionated via SDS-PAGE and transferred onto a polyvinylidene fluoride membrane. The membrane was incubated with antibodies against TGF-β, p62, Collagen1A1, phosphor-SMAD2/3, SMAD2/3, αSMA, LC3- II/I, phospho-p70S6K (Thr389), p70S6K, phospho-S6, S6, phospho-ERK, ERK, phospho-p38, p38, cyclinD1, GAPDH, and β-actin. The antibodies used for immunoblot analysis are listed in the Supplementary Materials and Methods. Densitometric analyses of immunoblotting bands were performed, and the ratios of phosphorylated-to-total protein or total protein-to-loading control are indicated in Figures S3 and S4, Table S2.

### 4.6. Autophagy Analysis

The autophagy detection kit (ENZ-KIT175, Enzo Life Sciences, Inc., Farmingdale, NY, USA) was used to analyze autophagy in LX-2 cells after a 12 h incubation with DMSO (negative control), 20 μM HNK, 10 μM CQ, or 2.5 μM Rapa according to the manufacturer’s instructions. A BZ-X800 microscope and its analyzer were used to assess the staining.

### 4.7. Apoptosis Detection Assay

An in situ apoptosis detection kit (Takara Bio, Shiga, Japan) was used for the analysis of LX-2 cells treated with HNK or vehicle according to the manufacturer’s instructions. Briefly, cells were incubated in chamber slides (SCS-N04, Matsunami Glass Ind., Osaka, Japan) with the indicated treatment for 24 h. The substrate 3,3′-diaminobenzidine (DAB) (Takara Bio, Shiga, Japan) was used for staining, which was observed with a BZ-X800 microscope. A total of 10 randomly selected fields of view at 200-fold magnification were used, and the number of the TUNEL-positive cells was averaged.

### 4.8. Small Interfering RNA (siRNA) Transfection

The siRNA used to downregulate expression of p38 MAPK was purchased from Ambion (Life Technologies, Carlsbad, CA, USA). LX2 cells (1 × 10^5^ cells/well) in 6-well plates were incubated overnight and then treated with Lipofectamine™ RNAiMAX transfection reagent (Life Technologies, Carlsbad, CA, USA) and Opti-MEM I (Life Technologies) with p38 MAPK siRNA or non-targeting siRNA control (Thermo Fisher Scientific, Pittsburgh, PA, USA) following the manufacturer’s instructions. After 24 h, the cell supernatant containing the transfection reagent was replaced with medium containing the indicated concentrations of HNK, and the cells were incubated for 24 h.

### 4.9. Statistical Analysis

Data are presented as the mean ± SD and were analyzed using the Wilcoxon signed-rank test (JMP8.0, SAS Institute Inc., Cary, NC, USA). *p* < 0.05 was considered significant.

## Data Availability

All data from the study are included in the article.

## Supplementary Materials

The following are available online at https://www.mdpi.com/article/10.3390/ijms222413354/s1.
