# Supplementary material for: Honokiol Acts as a Potent Anti-Fibrotic Agent in the Liver through Inhibition of TGF-β1/SMAD Signaling and Autophagy in Hepatic Stellate Cells
Source: Int J Mol Sci. 2021 Dec 12;22(24):13354. doi: 10.3390/ijms222413354 (PMC8705910; doi:10.3390/ijms222413354)
Supplement: Supplementary file 1 [file ijms-22-13354-s001.zip › ijms-1508900-supplementary.pdf]

Supplementary Figures and Tables

A

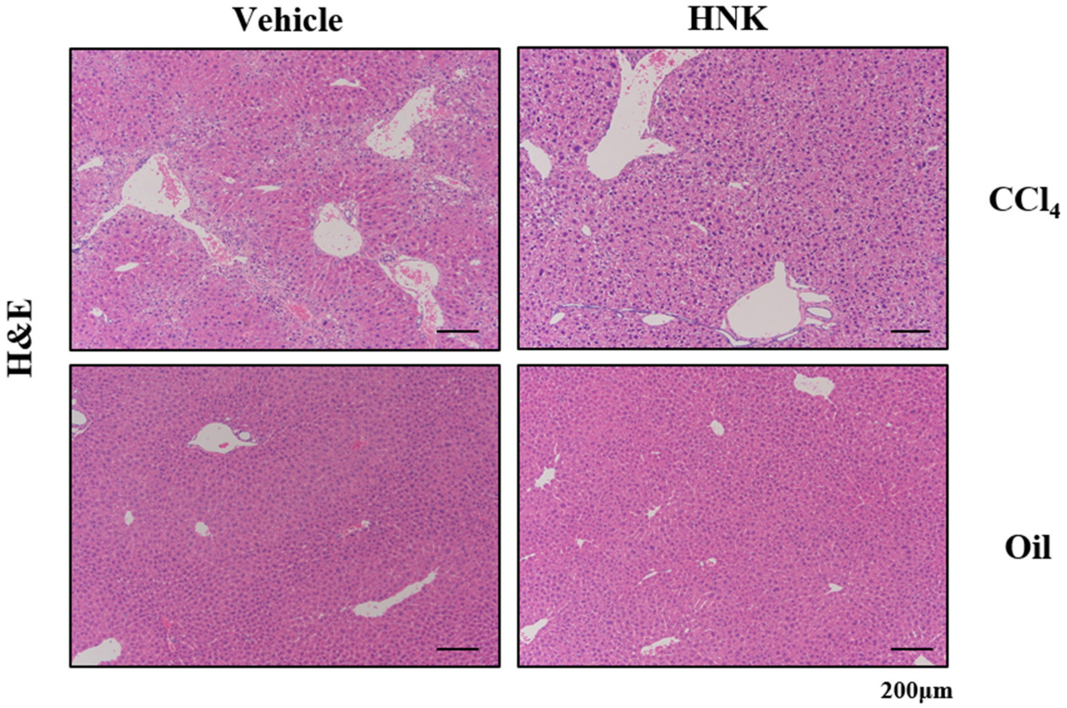

B

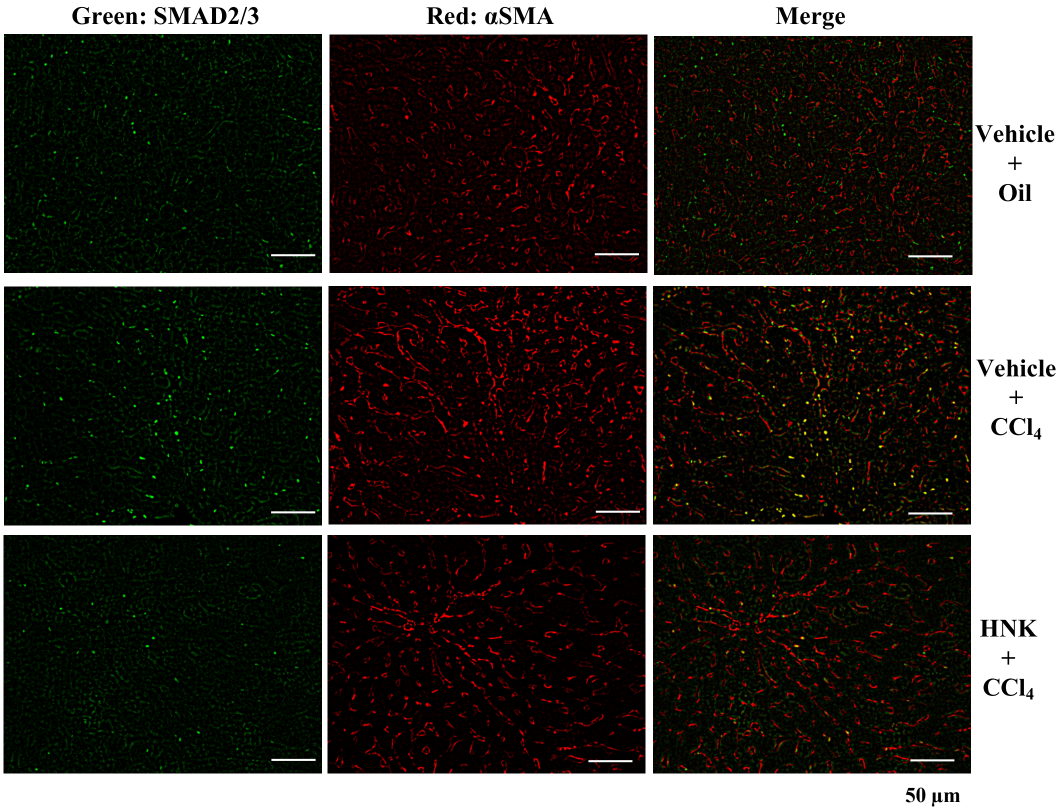
Green: SMAD2/3
Red: αSMA
Merge
Vehicle + Oil
Vehicle + CCl4
HNK + CCl4
50 µm

C

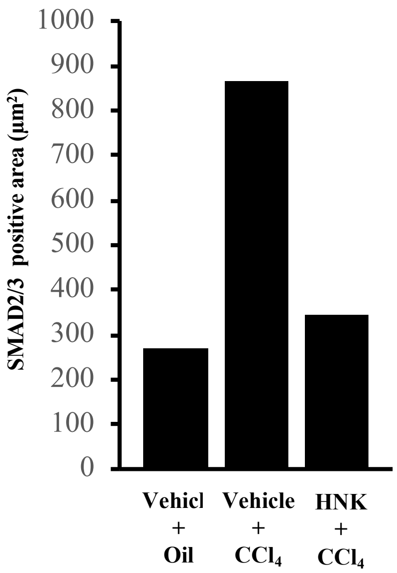

1000
900
800
700
600
500
400
300
200
100
0
SMAD2/3 positive area (μm²)
Vehicl + Oil
Vehicle + CCl4
HNK + CCl4

D

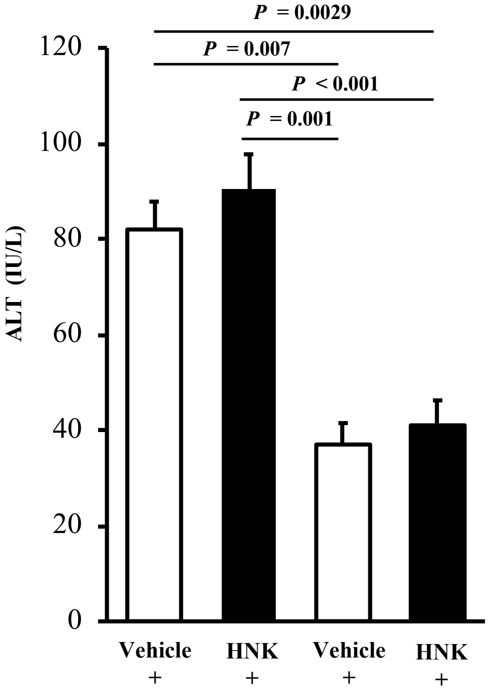

P = 0.0029
P = 0.007
P < 0.001
P = 0.001
120
100
80
60
40
20
0
ALT (IU/L)
Vehicle + CCl4
HNK + CCl4
Vehicle + Oil
HNK + Oil

E

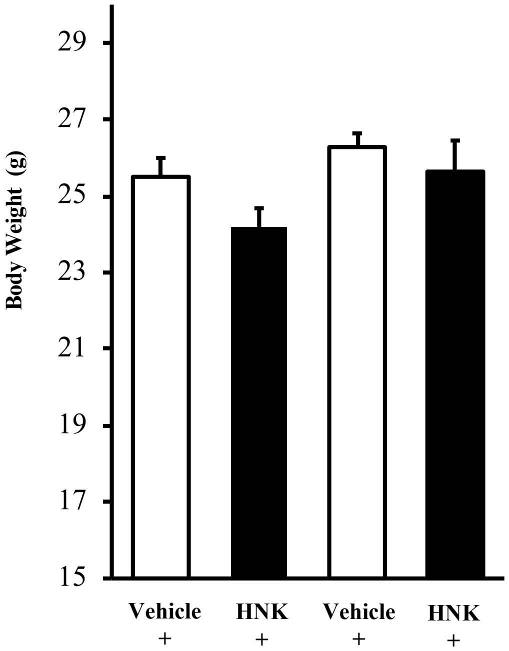

29
27
25
23
21
19
17
15
Body Weight (g)
Vehicle + CCl4
HNK + CCl4
Vehicle + Oil
HNK + Oil

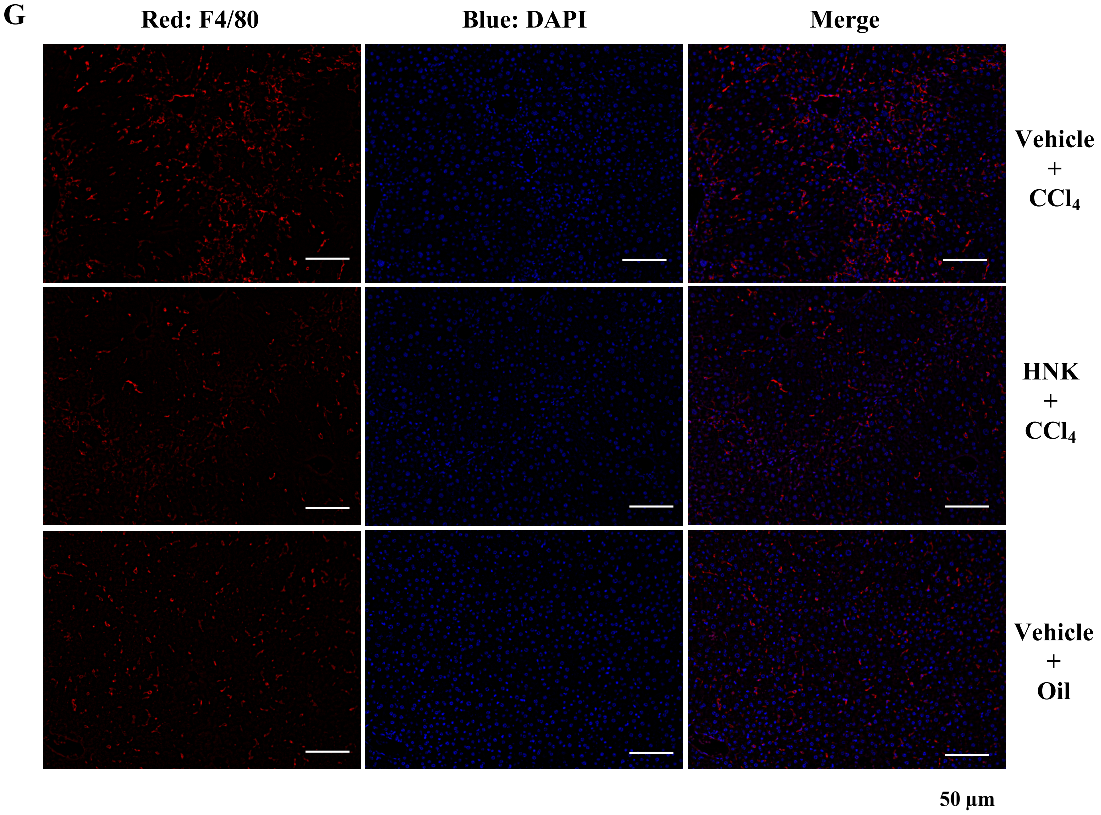

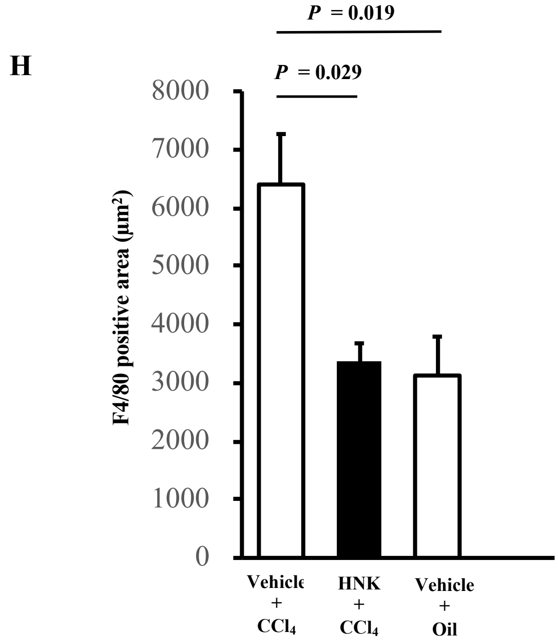

Figure S1. HNK treatment attenuates liver injury development and HSC activation in mice with CCl_4-induced liver injury, but no adverse effects were observed upon consideration of ALT levels and body weights. Mice treated with olive oil (at 5.0 μL/g body weight twice a week for 6 weeks from 10 to 16 weeks of age) instead of CCl_4 were also generated at the

same time as the CCl_4-treated group. Model and control animals were divided into two groups: the HNK-treated group (n = 5) and the vehicle-treated group (n = 5). Mice were injected with 20% CCl_4 at 5.0 µL/g body weight twice a week for 6 weeks (from 10 to 16 weeks of age). HNK at 10 µg/g body weight or vehicle control were administered three times per week on a day without CCl_4 or olive oil injections over the same six weeks. Liver injuries were assessed by HE staining and serum ALT analyses, and the attenuation of HSC activity and inflammation by HNK was assessed by immunofluorescence (IF). (A) Representative microphotographs of HE-stained liver sections. Scale bars represent 200 µm. (B) SMAD2/3 protein (green), α-Smooth muscle actin (αSMA) protein (red). Scale bars represent 50 µm. (C) SMAD2/3 positive area (µm^2) quantified by BZ-X800 Analyzer (D) ALT activity. CCl_4 + Vehicle group (n = 11): 82.0 ± 5.83 IU/L, CCl_4 + HNK group (n = 10): 90.4 ± 7.37 IU/L, Oil + Vehicle group (n = 5): 37 ± 4.51 IU/L, Oil + HNK group (n = 5): 41.0 ± 5.23 IU/L. (E) Body weight (g) at the time of analysis. Vehicle + CCl_4 group (n = 11): 25.5 ± 0.49 g, HNK + CCl_4 group (n = 10): 24.2 ± 0.50 g, Vehicle + Oil group (n = 5): 26.3 ± 0.36 g, HNK + Oil group (n = 5): 25.6 ± 0.81g. All graphs represent the mean ± SD.(F) F4/80 protein (red), DAPI (blue). Scale bars reprersent 50 µm. (G) F4/80 positive area (µm^2) quantified by BZ-X800 Analyzer.

A

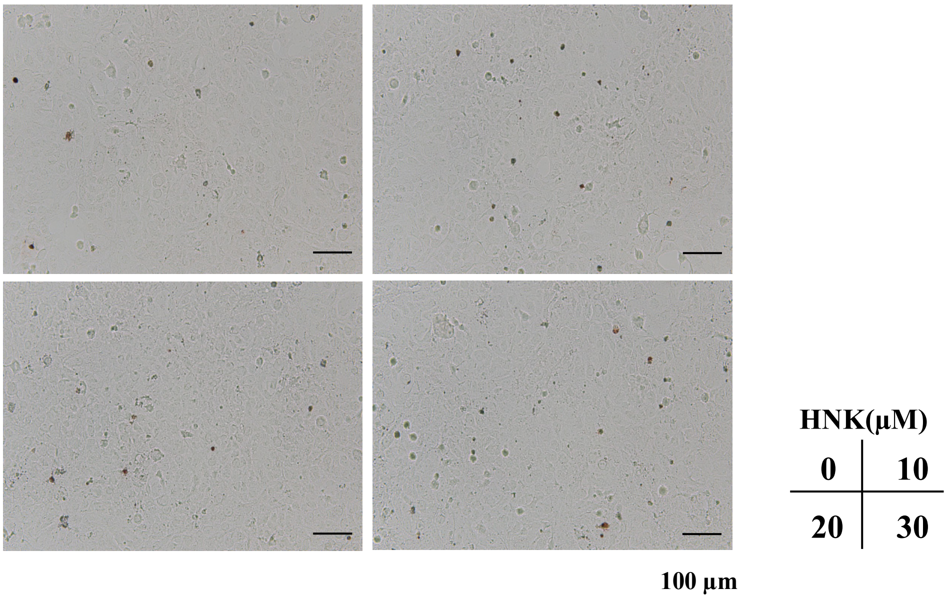

B

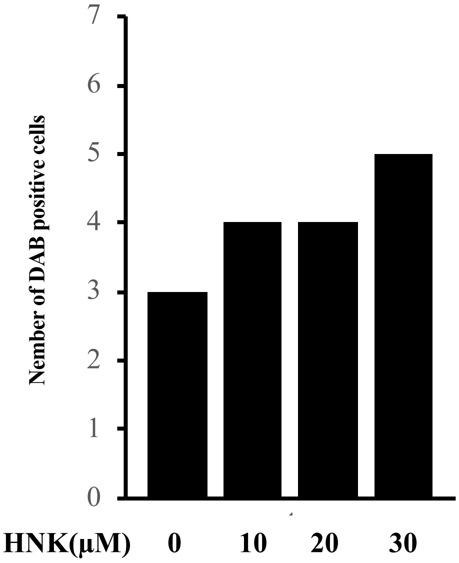

Figure S2. TUNEL staining of LX2 cells treated with HNK for 24 h. (A) LX2 cells were treated with HNK at the noted concentrations (μM) for 24 h, then the cells were reacted with Terminal deoxynucleotidyl transferase (TdT) and stained with 3,3′-diaminobenzidine (DAB). The samples were examined using fluorescence microscopy (BZ-X810, Keyence, Osaka, Japan). Scale bars, 100 µm. (B) The number of DAB-positive cells per field of view after treatment with HNK.

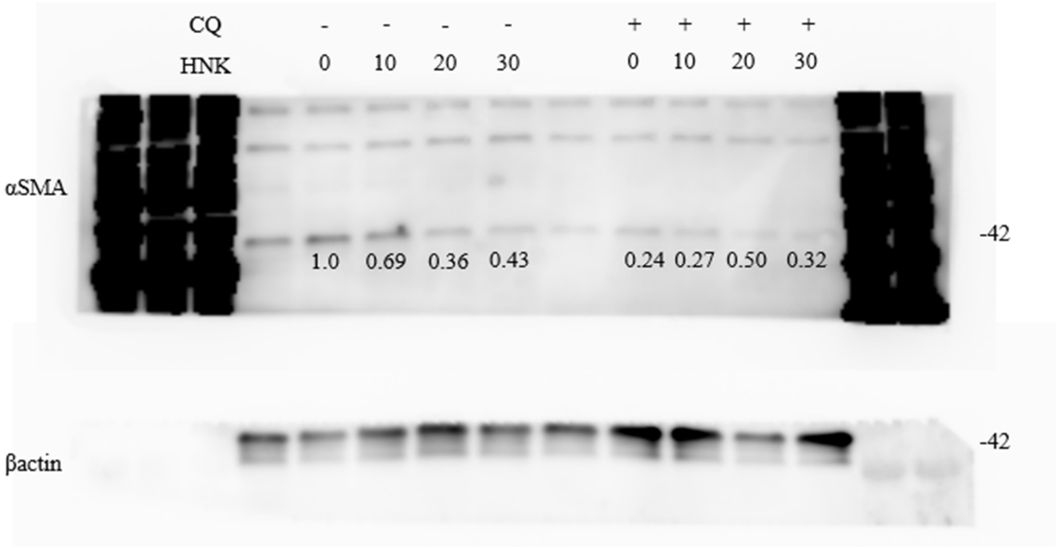

Figure S3 The suppressive effect of HNK on LX2 activation seems to be attenuated when HNK and CQ are co-administered. Immunoblotting was performed to access the HSC activation. After αSMA band intensities were determined by densitometry software, the loading control protein β-actin was used to normalize αSMA expression. To compare ratios of αSMA to β-actin across samples, the fold change was calculated by dividing the normalized expression from each lane by the normalized expression of the control sample (HNK 0, without CQ samples). The migrations of standard proteins of known molecular weight (kDa) are displayed to the right of the blots.

Fig S4.

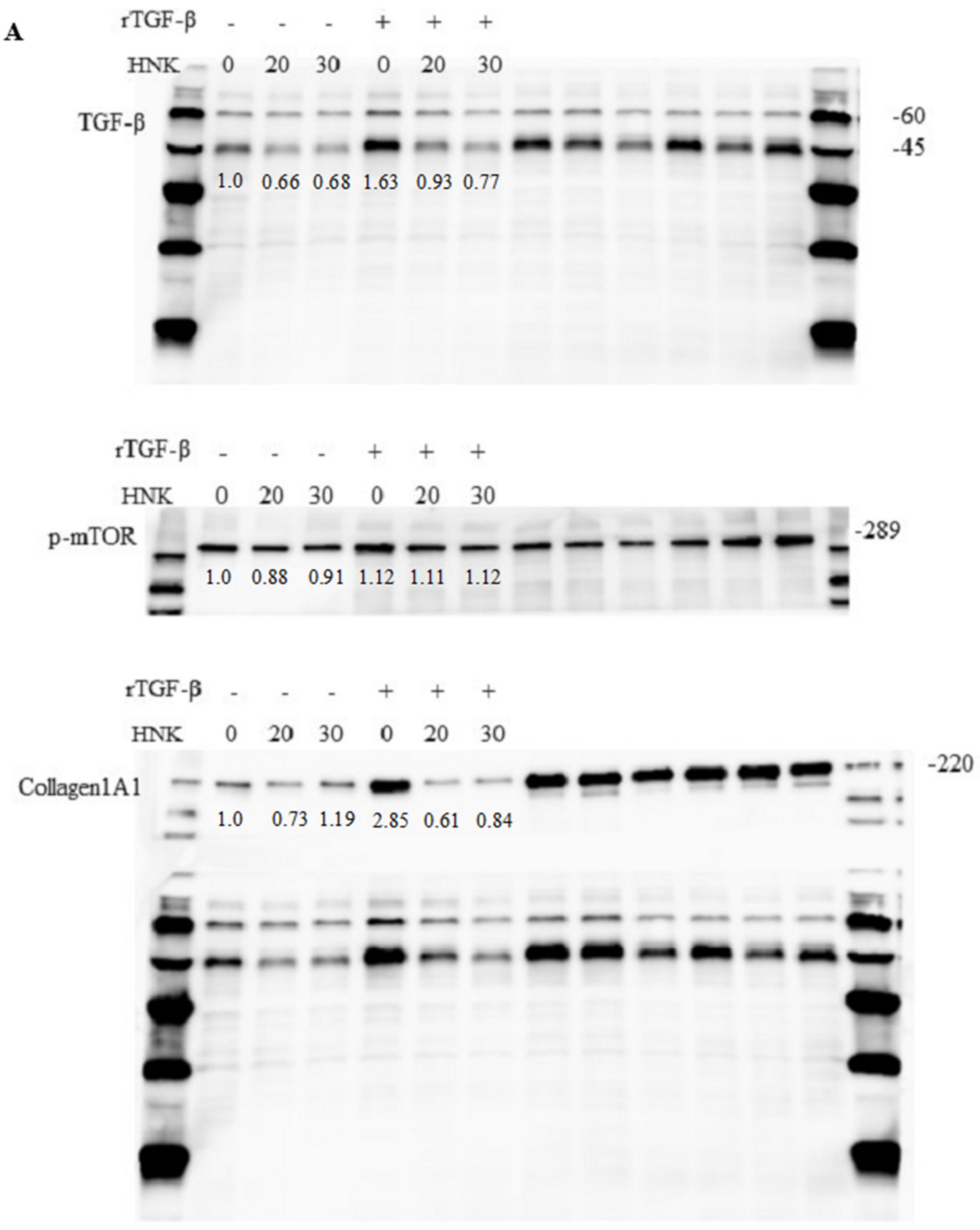
A
rTGF-β - - - + + +
HNK 0 20 30 0 20 30
TGF-β
-60
-45
1.0 0.66 0.68 1.63 0.93 0.77
rTGF-β - - - + + +
HNK 0 20 30 0 20 30
p-mTOR
-289
1.0 0.88 0.91 1.12 1.11 1.12
rTGF-β - - - + + +
HNK 0 20 30 0 20 30
Collagen1A1
-220
1.0 0.73 1.19 2.85 0.61 0.84

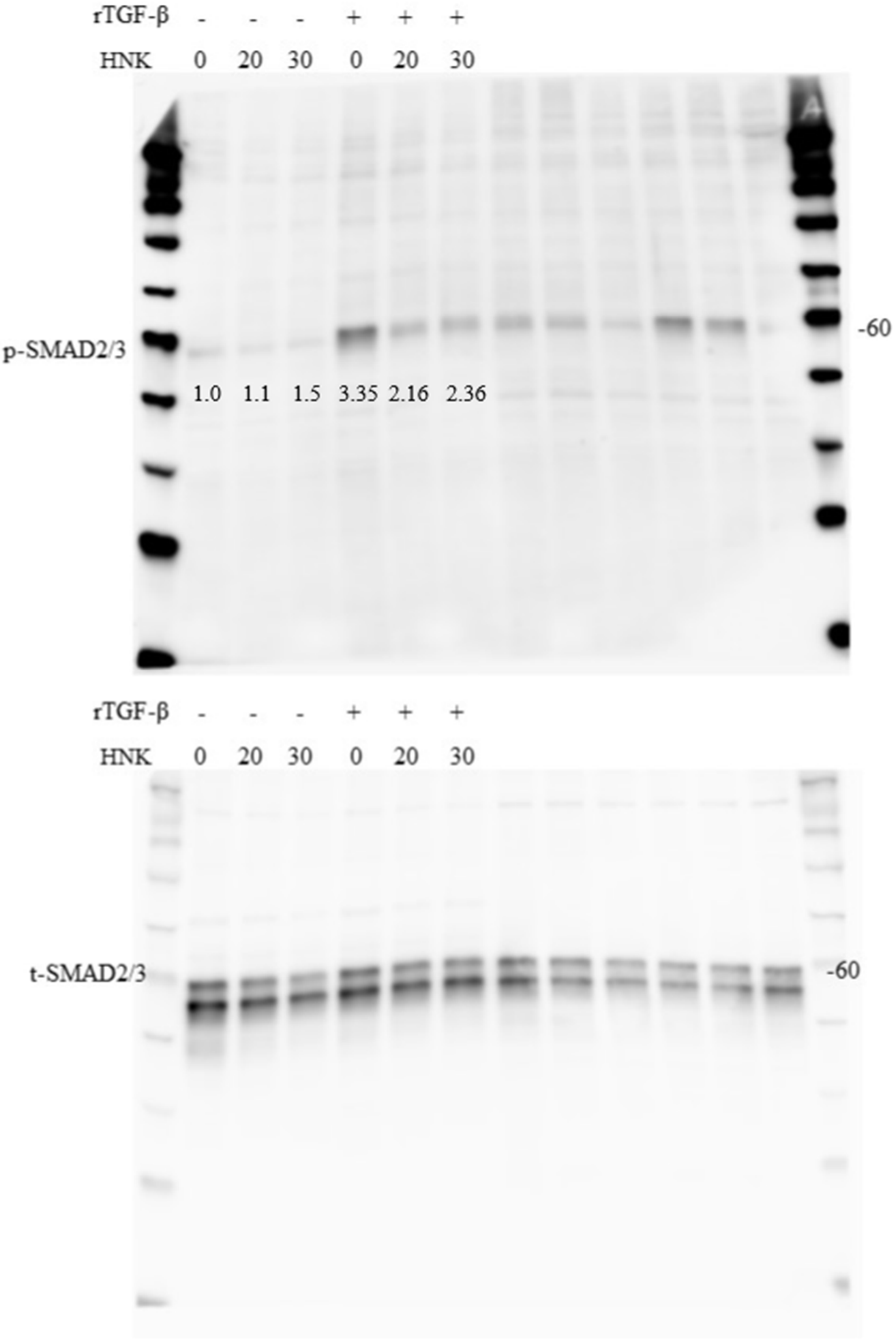
rTGF-β - - - + + +
HNK 0 20 30 0 20 30
p-SMAD2/3
-60
1.0 1.1 1.5 3.35 2.16 2.36
rTGF-β - - - + + +
HNK 0 20 30 0 20 30
t-SMAD2/3
-60

rTGF-β - - - + + +

HNK 0 20 30 0 20 30

p-p38

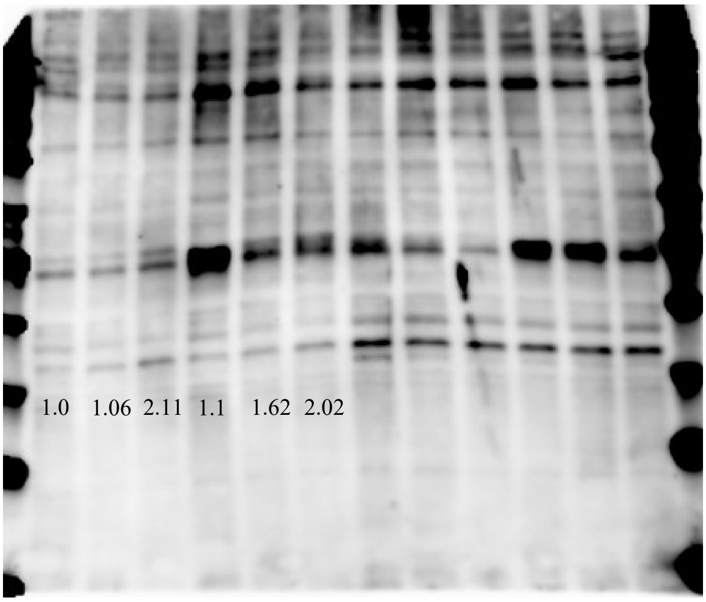

-38

t-p38

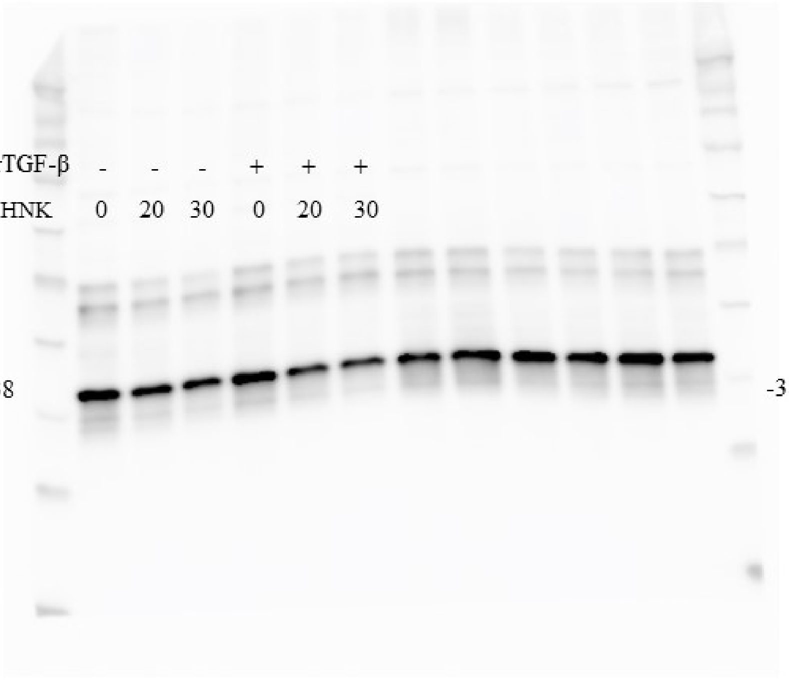

-38

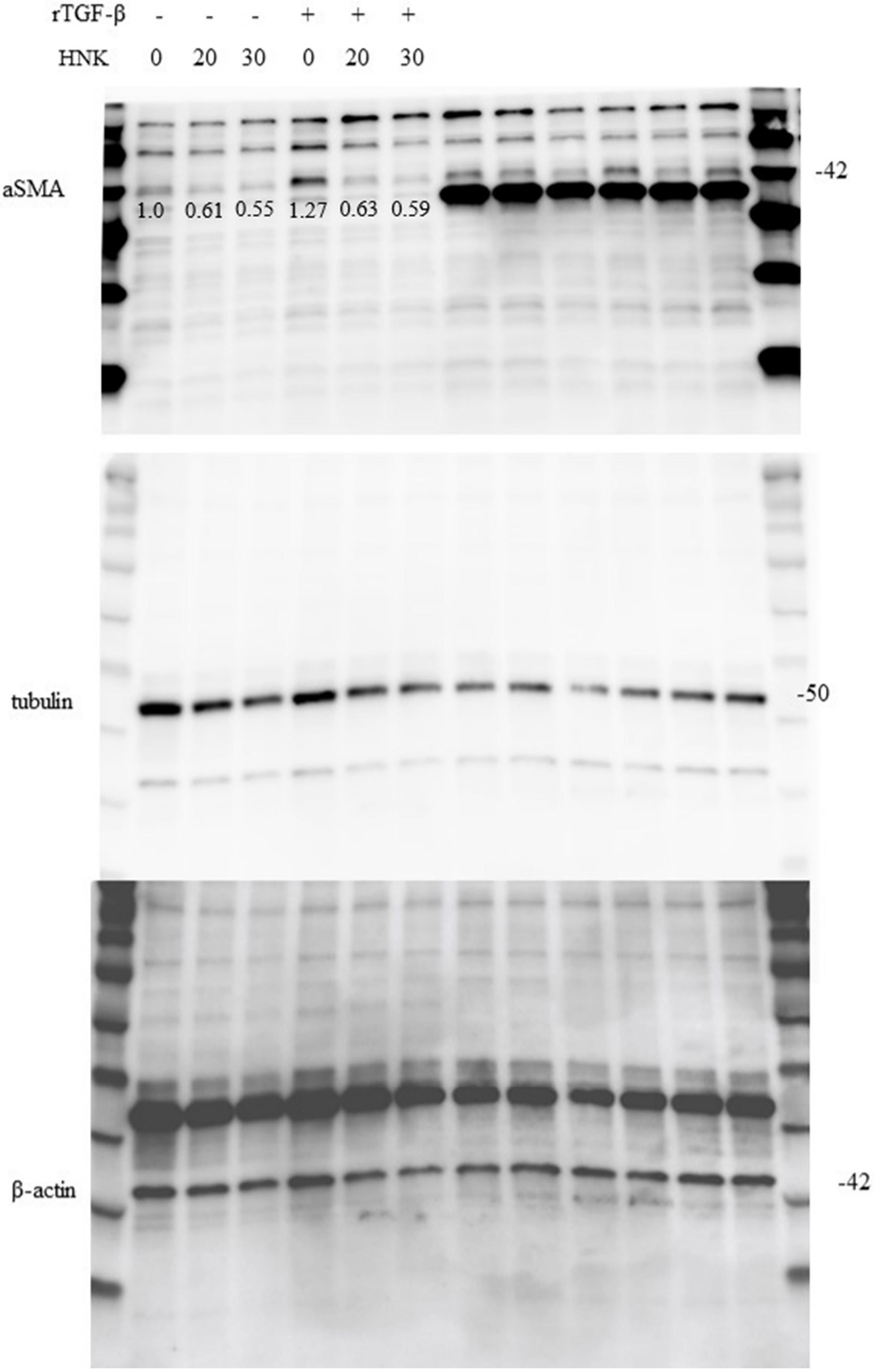

rTGF-β - - - + + +
HNK 0 20 30 0 20 30
aSMA
1.0 0.61 0.55 1.27 0.63 0.59
-42
tubulin
-50
β-actin
-42

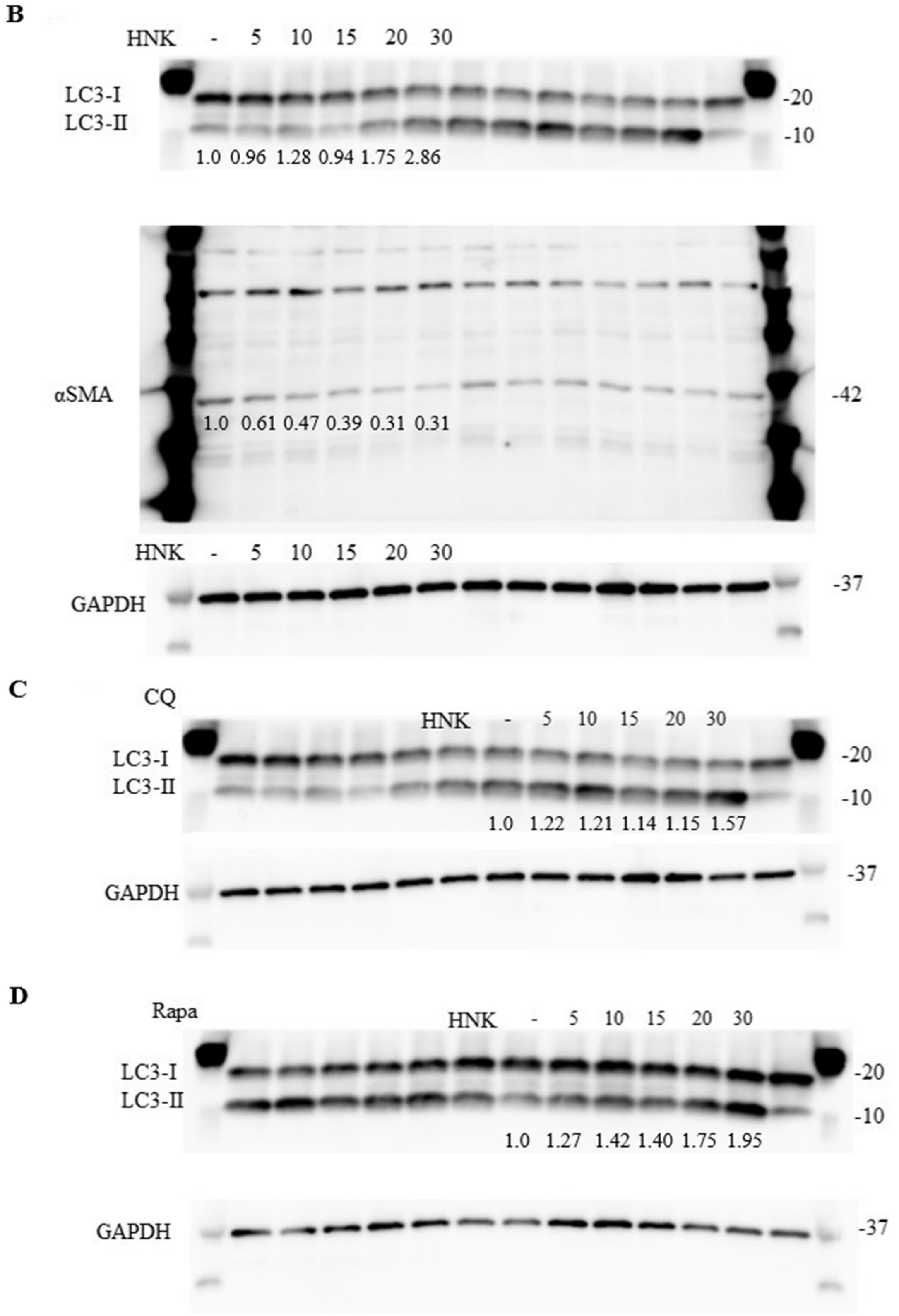
B
HNK - 5 10 15 20 30
LC3-I
LC3-II
-20
-10
1.0 0.96 1.28 0.94 1.75 2.86
αSMA
-42
1.0 0.61 0.47 0.39 0.31 0.31
HNK - 5 10 15 20 30
GAPDH
-37
C
CQ
HNK - 5 10 15 20 30
LC3-I
LC3-II
-20
-10
1.0 1.22 1.21 1.14 1.15 1.57
GAPDH
-37
D
Rapa
HNK - 5 10 15 20 30
LC3-I
LC3-II
-20
-10
1.0 1.27 1.42 1.40 1.75 1.95
GAPDH
-37

E

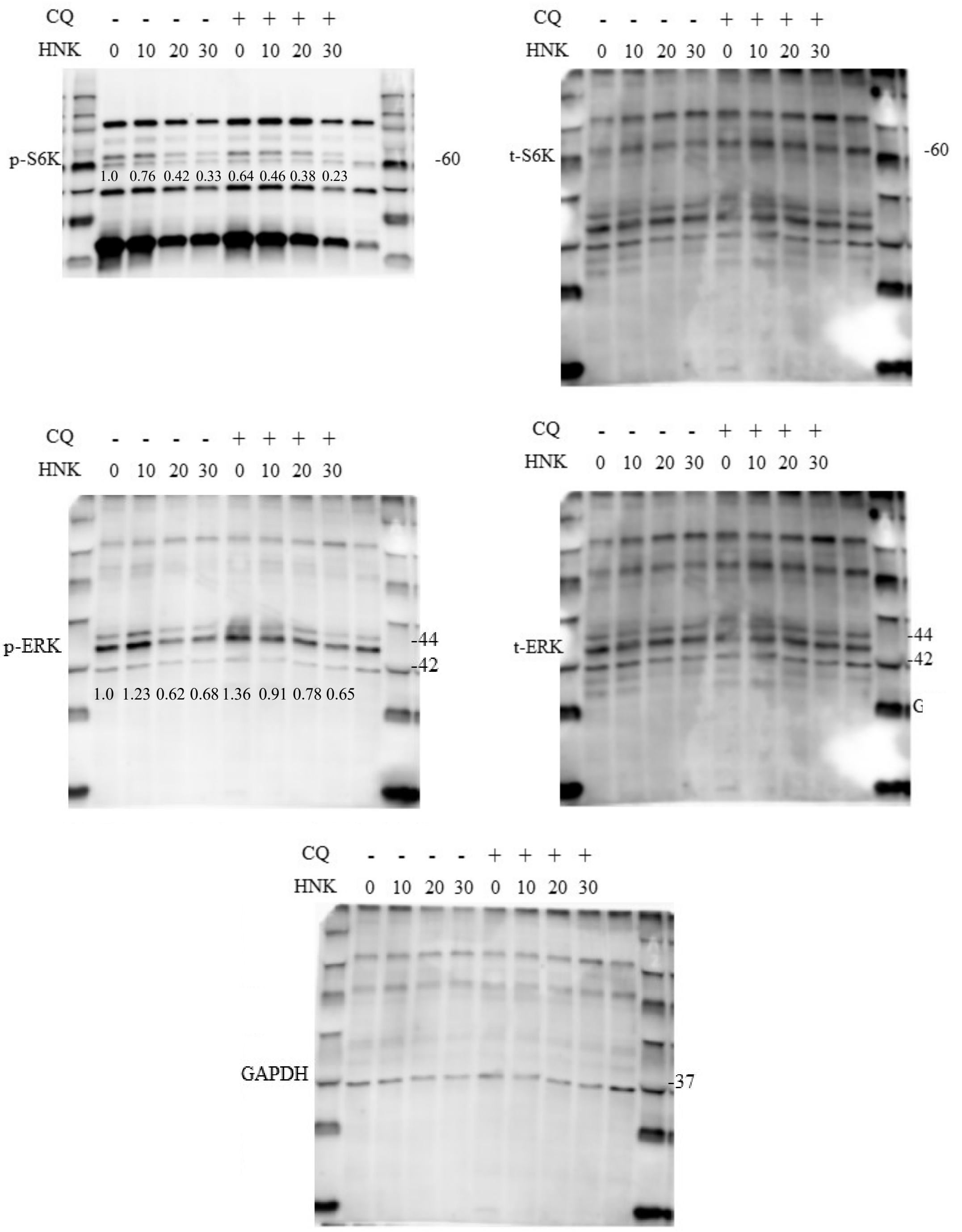
CQ - - - - + + + +
HNK 0 10 20 30 0 10 20 30
p-S6K
1.0 0.76 0.42 0.33 0.64 0.46 0.38 0.23
-60
CQ - - - - + + + +
HNK 0 10 20 30 0 10 20 30
t-S6K
-60
CQ - - - - + + + +
HNK 0 10 20 30 0 10 20 30
p-ERK
-44
-42
1.0 1.23 0.62 0.68 1.36 0.91 0.78 0.65
CQ - - - - + + + +
HNK 0 10 20 30 0 10 20 30
t-ERK
-44
-42
CQ - - - - + + + +
HNK 0 10 20 30 0 10 20 30
GAPDH
-37

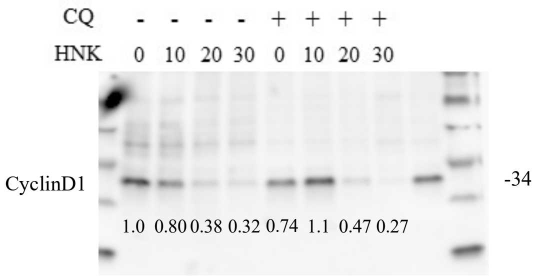
CQ - - - - + + + +
HNK 0 10 20 30 0 10 20 30
CyclinD1
-34
1.0 0.80 0.38 0.32 0.74 1.1 0.47 0.27

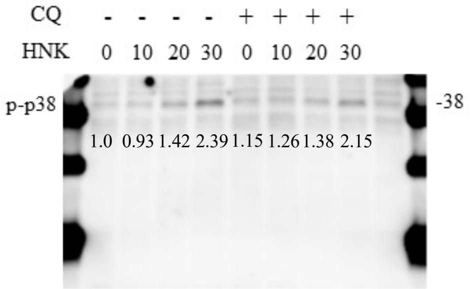
CQ - - - - + + + +
HNK 0 10 20 30 0 10 20 30
p-p38
-38
1.0 0.93 1.42 2.39 1.15 1.26 1.38 2.15

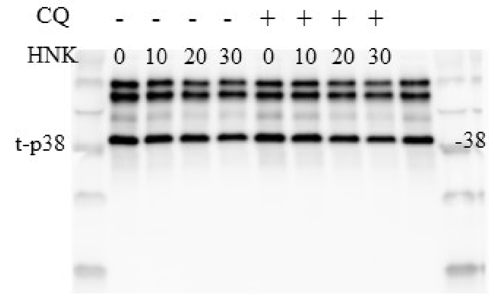
CQ - - - - + + + +
HNK 0 10 20 30 0 10 20 30
t-p38
-38

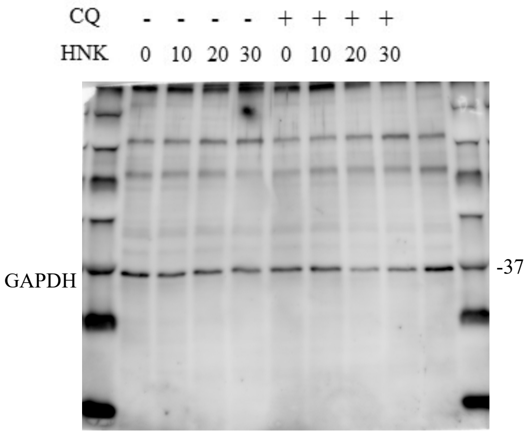
CQ - - - - + + + +
HNK 0 10 20 30 0 10 20 30
GAPDH
-37

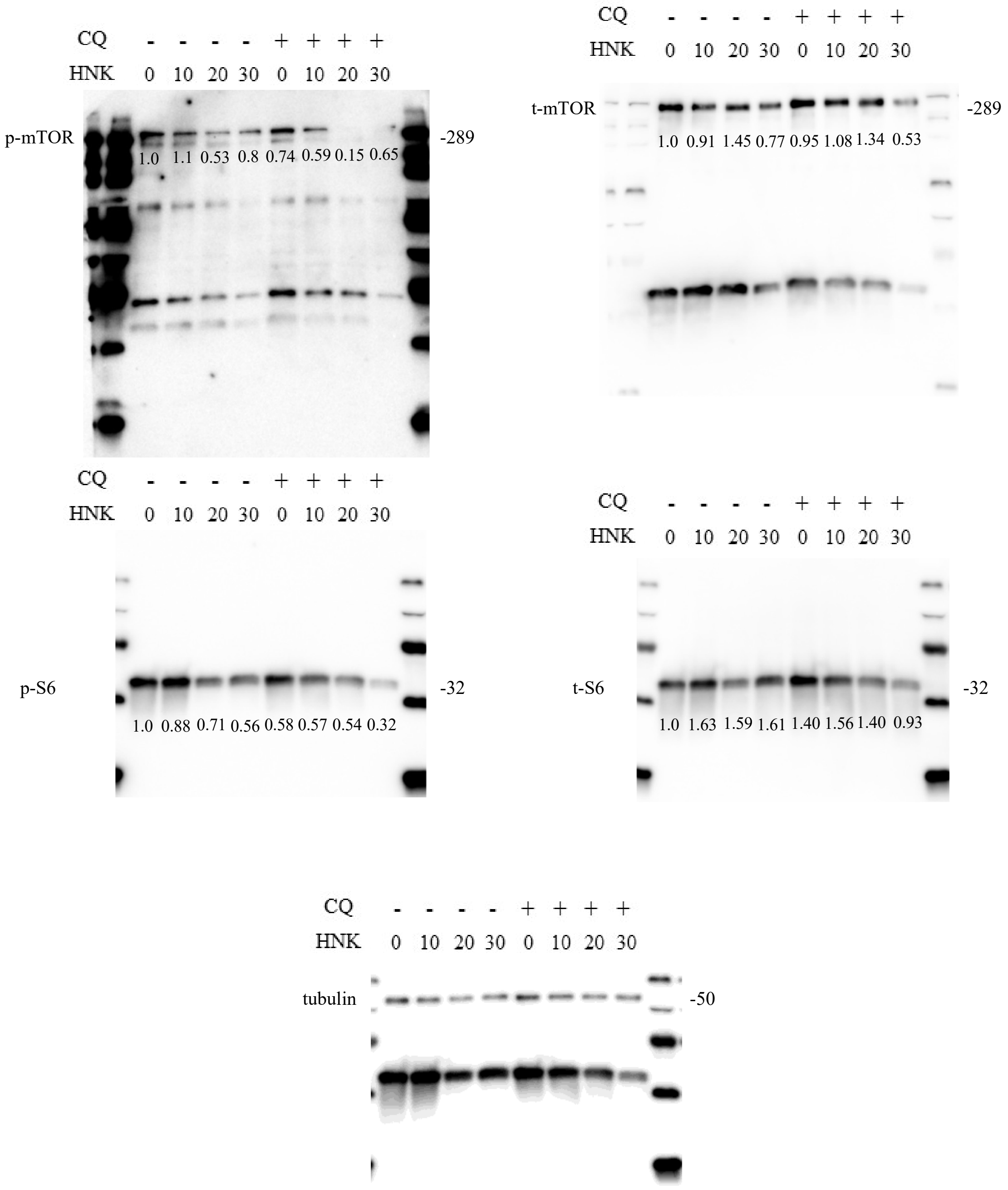

CQ - - - - + + + +
HNK 0 10 20 30 0 10 20 30
p-mTOR
-289
1.0 1.1 0.53 0.8 0.74 0.59 0.15 0.65
CQ - - - - + + + +
HNK 0 10 20 30 0 10 20 30
t-mTOR
-289
1.0 0.91 1.45 0.77 0.95 1.08 1.34 0.53
CQ - - - - + + + +
HNK 0 10 20 30 0 10 20 30
p-S6
-32
1.0 0.88 0.71 0.56 0.58 0.57 0.54 0.32
CQ - - - - + + + +
HNK 0 10 20 30 0 10 20 30
t-S6
-32
1.0 1.63 1.59 1.61 1.40 1.56 1.40 0.93
CQ - - - - + + + +
HNK 0 10 20 30 0 10 20 30
tubulin
-50

F

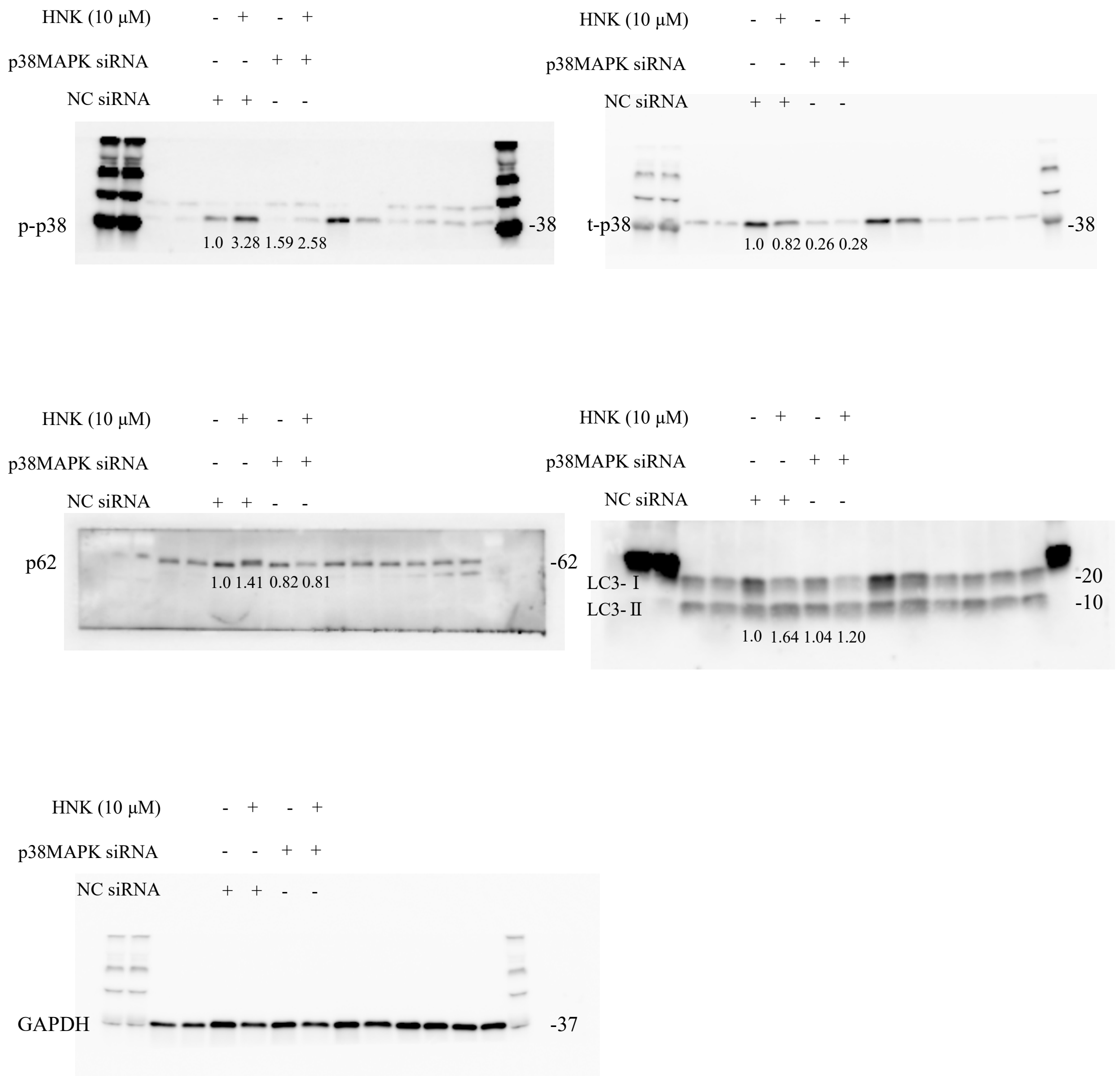

Figure S4. Original images for immunoblotting analyses. Densitometric analysis of bands was performed, and the ratios of phosphorylated–total protein or total protein–loading control are calculated. After band intensities were determined by densitometry software, the loading control protein (or the total protein in case of phosphorylated protein) is used to normalize target protein expression. To compare relative target protein levels across samples, the fold change can then be calculated by dividing the normalized expression from each lane by the normalized expression of the control sample (HNK 0, without any treatment sample).

(A) The ratios of TGF-β, p-mTOR, Collagen1A1, and αSMA related to β-actin

presented under the bands. These images support Fig. 4 in the manuscript.

(B) (C) (D) GAPDH was used as loading controls for αSMA. The numbers under each band (LC3) indicate the ratio of LC3-II to LC3-I (LC3-II/LC3-I). These images support Fig. 5 (A), (B) and (C) in the manuscript.

(E) Protein expression and phosphorylation of mTOR, MAPK signaling-related molecules are presented. GAPDH and tubulin was used as a loading control. These images support Fig. 6 in the manuscript.

(F) p-p38 MAPK, t-p38 MAPK, LC3 and p62 levels in LX-2 cells transfected with p38 MAPK or negative control (NC) siRNA and further treated with HNK for 24 h. To compare ratios of LC3- Ⅱ or p62 to GAPDH across samples, the fold change was calculated by dividing the normalized expression from each lane by the normalized expression of the control sample (HNK 0, with NC siRNA)

Table S1. Primers used for quantitative RT-PCR

**Species**	**Target Gene**	**Sequence (5' - 3')**	
human	*ACTA2*	forward	GGCAGTGGCCATCTCATTTTC
human	*ACTA2*	reverse	GCGTGGCTATTCCTTCGTTAC
human	*COLLAGEN1A1*	forward	GATTCCCTGGACCTAAAGGTGC
human	*COLLAGEN1A1*	reverse	AGCCTCTCCATCTTTGCCAGCA
human	*COLLAGEN3A1*	forward	AGAGACCTGAAATTCTGCCATCC
human	*COLLAGEN3A1*	reverse	GCATGTTTCCCCAGTTTCCA
human	*GAPDH*	forward	TGACAACTTTGGTATCGTGGAAGG
human	*GAPDH*	reverse	AGGCAGGGATGATGTTCTGGAGAG
human	*GUSB*	forward	GTCTGCGGCATTTTGTCGG
human	*GUSB*	reverse	CACACGATGGCATAGGAATGG
mouse	*Collagen1a1*	forward	GCTCCTCTTAGGGGCCACT

mouse	*Collagen1a1*	reverse	CCACGTCTCACCATTGGGG
mouse	*Gapdh*	forward	AACTTTGGCATTGTGGAAGG
mouse	*Gapdh*	reverse	CCCTGTTGCTGTAGCCGTAT

Table S2. Antibodies for immunoblotting

Target	Catalog#	Company
TGFβ	3709	Cell signaling technology
αSMA	Ab5694	Abcam
COLLAGEN1A1	91144	Cell signaling technology
SMAD2/3	8685	Cell signaling technology
p-SMAD2 (Ser465/467) /3 (Ser423/425)	8828	Cell signaling technology
P62	GP62-C	Progen
LC3	2775	Cell signaling technology
p-mTOR	2971	Cell signaling technology
mTOR	2972	Cell signaling technology
ERK	4695	Cell signaling technology
p-ERK	9101	Cell signaling technology
P38	8690	Cell signaling technology
p-P38	4370	Cell signaling technology
P70S6K	9202	Cell signaling technology
p-P70S6K(THR389)	9205	Cell signaling technology
S6	2217	Cell signaling technology
p-S6	4858	Cell signaling technology
CyclinD1	2978	Cell signaling technology

β-actin	A1978	Sigma-Aldrich
Tubulin	T9026	Sigma-Aldrich
GAPDH	5174	Cell signaling technology
Anti-rabbit IgG, HRP-linked	7074	Cell signaling technology
Anti-mouse IgG, HRP-linked	7076	Cell signaling technology
Anti-goat IgG, HRP-linked	J0710	Santa Cruz Biotechnology

Supplementary Materials and Methods

Animals and liver analysis

After mice were sacrificed, their livers were removed and separated into individual lobes. Externally visible tumors (>1 mm) were counted and measured. Large lobes were fixed in 10% formalin for 24 to 48 hours for paraffin block preparation, respectively. Paraffin-embedded liver tissues were used for HE and Sirius Red staining. Sirius Red staining (Sirius Red F3B, catalog number 365548, Sigma-Aldrich) was performed to quantify the amount of collagen fibers. Sirius Red-positive areas were counted in 6 to 10 random fields at ×100 magnification on each slide [1]. All quantification results are depicted in the bar graphs. Remaining lobes were micro-dissected into tumor and nontumor tissue and stored at -80 °C until analyzed.

Supplementary References

1. Umemura A, Park EJ, Taniguchi K, Lee JH, Shalapour S, Valasek MA*, et al.* Liver

damage, inflammation, and enhanced tumorigenesis after persistent mTORC1 inhibition. Cell Metab **2014**;20:133–144
